# Type One Protein Phosphatase 1 and Its Regulatory Protein Inhibitor 2 Negatively Regulate ABA Signaling

**DOI:** 10.1371/journal.pgen.1005835

**Published:** 2016-03-04

**Authors:** Yueh-Ju Hou, Yingfang Zhu, Pengcheng Wang, Yang Zhao, Shaojun Xie, Giorgia Batelli, Bangshing Wang, Cheng-Guo Duan, Xingang Wang, Lu Xing, Mingguang Lei, Jun Yan, Xiaohong Zhu, Jian-Kang Zhu

**Affiliations:** 1 Department of Horticulture and Landscape Architecture, Purdue University, West Lafayette, Indiana, United States of America; 2 Shanghai Center for Plant Stress Biology and Shanghai Institute of Plant Physiology and Ecology, Shanghai Institutes of Biological Sciences, Chinese Academy of Sciences, China; 3 Shanghai Center for Plant Stress Biology, Shanghai Institutes of Biological Sciences, Chinese Academy of Sciences, China; National University of Singapore and Temasek Life Sciences Laboratory, SINGAPORE

## Abstract

The phytohormone abscisic acid (ABA) regulates plant growth, development and responses to biotic and abiotic stresses. The core ABA signaling pathway consists of three major components: ABA receptor (PYR1/PYLs), type 2C Protein Phosphatase (PP2C) and SNF1-related protein kinase 2 (SnRK2). Nevertheless, the complexity of ABA signaling remains to be explored. To uncover new components of ABA signal transduction pathways, we performed a yeast two-hybrid screen for SnRK2-interacting proteins. We found that Type One Protein Phosphatase 1 (TOPP1) and its regulatory protein, At Inhibitor-2 (AtI-2), physically interact with SnRK2s and also with PYLs. TOPP1 inhibited the kinase activity of SnRK2.6, and this inhibition could be enhanced by AtI-2. Transactivation assays showed that TOPP1 and AtI-2 negatively regulated the SnRK2.2/3/6-mediated activation of the ABA responsive reporter gene *RD29B*, supporting a negative role of TOPP1 and AtI-2 in ABA signaling. Consistent with these findings, *topp1* and *ati-2* mutant plants displayed hypersensitivities to ABA and salt treatments, and transcriptome analysis of *TOPP1* and *AtI-2* knockout plants revealed an increased expression of multiple ABA-responsive genes in the mutants. Taken together, our results uncover TOPP1 and AtI-2 as negative regulators of ABA signaling.

## Author Summary

The phytohormone abscisic acid (ABA) regulates multiple developmental processes such as seed dormancy, germination, root/shoot growth, flowering and senescence in plants. Although the core ABA perception and signaling pathway has been elucidated, the complexity of the pathway remains to be exploited. In the present work, we uncovered two new proteins, TOPP1 and its regulatory protein AtI-2, interact with both ABA receptor PYLs and their downstream positive regulator SnRK2s. In addition to their physical interaction, TOPP1 could inhibit the kinase activity of SnRK2s and this inhibition could be further enhanced by AtI-2, which is likely due to a promotion of the interaction between TOPP1 and SnRK2s by AtI-2. *topp1* and *ati-2* mutants exhibited hypersensitivity to ABA and salt treatments; and transcriptome studies revealed multiple ABA-responsive genes were up-regulated in the mutants. In summary, our work identified two new components, TOPP1 and AtI-2, and characterized their negative roles in ABA signaling.

## Introduction

The phytohormone abscisic acid (ABA) in plants controls a variety of developmental processes such as seed dormancy, germination, root/shoot growth, flowering and senescence [1–3]. When plants encounter stressful conditions, ABA can rapidly induce the reprogramming of gene expression and trigger multiple physiological responses such as stomatal closure to reduce water loss in plants [4–6]. Given the importance of ABA in regulating various aspects of plant growth and stress responses, it is critical to understand the molecular mechanisms of ABA action in response to adverse environmental conditions.

*Arabidopsis ABA insensitive 1* (*abi1-1*) and *abi2-1* mutants were isolated in genetic screens for ABA insensitivity phenotypes [7]. Both mutants showed dominant-negative effects during seed germination, seedling growth and stomatal closure. The discovery that *ABI1* and *ABI2* encode homologous type 2C Serine/Threonine (Ser/Thr) phosphatases (PP2Cs) revealed critical roles for phosphatase-mediated dephosphorylation in regulating ABA signaling ([8] [9] [10]). It has been shown that these clade A PP2Cs negatively regulate the ABA signal transduction pathway [11].

The SNF1-related protein kinases (SnRK2s) also regulate ABA signaling. The *Arabidopsis* mutant *ost1-1/snrk2*.*6* (*Open Stomata 1*, also known as *SnRK2*.*6*) displays impaired ABA-induced stomatal closure and defective light-induced stomatal opening [12]. Ten SnRK2 members (SnRK2.1–10) have been identified in *Arabidopsis* [13]; however only four (SnRK2.2/2.3/2.6/2.8) can be activated by ABA in a protoplast transient expression assay, implying that SnRK2 members may function in both ABA-dependent and independent signaling pathways [13,14]. ABA-induced gene expression and other ABA responses were almost completely blocked in *snrk2*.*2/3/6* triple mutant plants [15–17], demonstrating that these three SnRK2s are critical positive regulators of ABA signaling. It was shown that the clade A PP2Cs bind to the SnRK2s and dephosphorylate key Ser/Thr residues in the activation loop of SnRK2s [18,19].

In recent years, ABA receptors were identified as the *PY**RABACTIN* *R**ESISTANCE 1* (PYR1)/PYR1-Like (PYL)/REGULATORY COMPONENT OF ABA RECEPTOR (RCAR) family of proteins [20,21]. ABA binding to the receptors triggers a conformational change in the receptors, allowing them to associate with PP2Cs and thus disrupting the PP2C-SnRK2 interactions and releasing the SnRK2s from PP2Cs-mediated inhibition [20–26]. Activated SnRK2s subsequently phosphorylate downstream target proteins such as ion channels in the plasma membrane and basic-domain leucine zipper (bZIP) transcription factors in the nucleus [4–6,27].

To identify potential new components of the ABA signaling pathway, we performed a yeast two-hybrid screen employing SnRK2.6 as bait. We discovered that Type One Phosphatase 1 (TOPP1) and its regulatory protein, *Arabidopsis* Inhibitor-2 (AtI-2), interacted with several SnRK2s and PYLs. Biochemical and physiological evidence indicates that TOPP1 and AtI-2 coordinately inactivate SnRK2s and, in turn, negatively regulate the ABA signal transduction pathway. *topp1* and *ati-2* mutant plants are hypersensitive to ABA and salt, consistent with their function in ABA signaling. Furthermore, transcriptome analysis revealed that TOPP1 and AtI-2 co-regulate groups of overlapping genes in response to ABA. Taken together, our work identifies new components of the ABA signaling pathway.

## Results

### TOPP1 and its regulatory protein AtI-2 physically interact with several SnRK2s

To identify potential new components in early ABA signaling, we used SnRK2.6 as bait to find interacting proteins in a yeast two-hybrid (Y2H) screen. Full-length SnRK2.6 cDNA was fused to the yeast GAL4 DNA Binding Domain (BD). A yeast strain carrying the bait construct was transformed with a cDNA plasmid library in which the cDNAs were fused to the GAL4 DNA Activation Domain (AD). One of the potential interactions identified was between TYPE ONE PROTEIN PHOSPHATASE 1 (TOPP1) and SnRK2.6.

**Fig 1:**
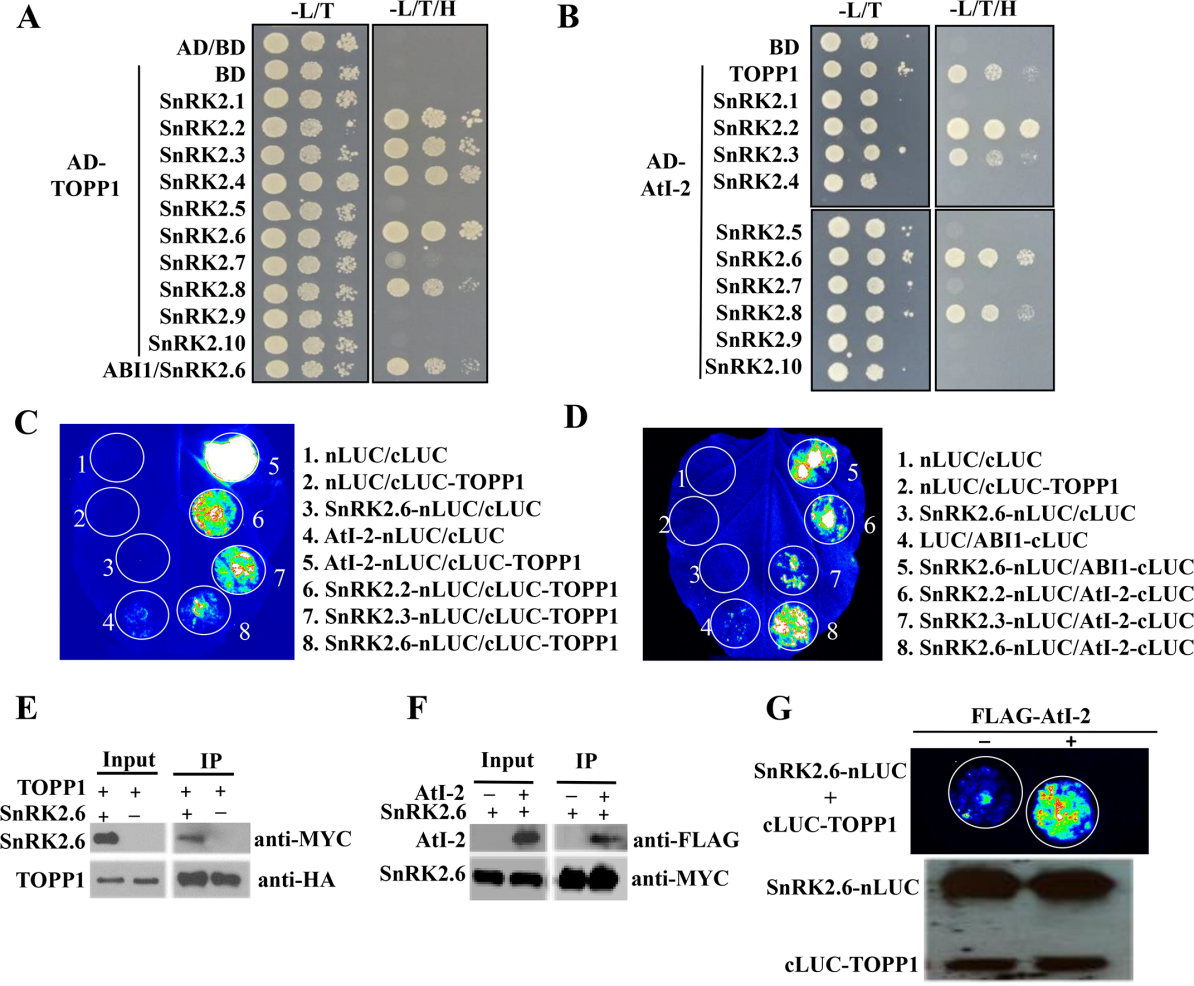
TOPP1 and AtI-2 physically interact with SnRK2s. (A) TOPP1 interacts with SnRK2s in Y2H assay. (B) AtI-2 interacts with SnRK2s in Y2H assay. TOPP1 or AtI-2 was fused to the GAL4-activating domain (AD) and SnRK2s were fused to the GAL4-DNA binding domain (BD). Interaction in Y2H assay was determined by yeast growth on media lacking Leu, Trp and His in the absence or presence of ABA. Dilutions (10^−1^, 10^−2^ and 10^−3^) of saturated cultures were spotted onto the plates and photographs were taken after 3 days. (C) TOPP1 interacts with SnRK2s in split-luciferase complementation (split-LUC) assay. (D) AtI-2 interacts with SnRK2s in split-LUC assay. (E) TOPP1 interacts with SnRK2.6 in Co-IP assay. (F) AtI-2 interacts with SnRK2.6 in Co-IP assay. (G) Alt-2 enhances the interaction between TOPP1 and SnRK2.6 in split-LUC assay. The split-LUC assays were repeated at least three times. Anti-LUC western blot shows the levels of the indicated fusion proteins.

We cloned the full-length coding sequence of *TOPP1* to validate and extend the potential interaction between TOPP1 and ten members of the SnRK2 family. Yeast co-expressing AD-TOPP1 and several members of SnRK2 family (SnRK2.2/3/4/6/8) fused to the BD could grow on the selection medium as well as the positive control that co-expressed AD-ABI1 and BD-SnRK2.6 (Fig 1A), indicating that TOPP1 interacts with the SnRK2s. Similarly, Y2H assays suggested that TOPP2 also interacts with SnRK2.2 and SnRK2.6, out of several SnRK2s tested (S1 Fig).

From data mining of SnRK2-interacting proteins within the *Arabidopsis thaliana* Protein Interaction Network [28], we noticed that ARABIDOPSIS Protein Phosphatase Inhibitor-2 (AtI-2) was predicted to interact with SnRK2.6. To evaluate potential interactions between AtI-2 and SnRK2s, we cloned the full-length coding sequence of AtI-2 and fused it to the AD vector. Consistent with previous report (Templeton *et al*, 2011), we found that AtI-2 interacted with TOPP1 in the Y2H assay. In addition, yeast co-transformed with AD-AtI-2 and several BD-SnRK2s (SnRK2.2/3/6/8) also grew on the selection medium (Fig 1B*)*. Taken together, our Y2H results indicated that both TOPP1 and its regulatory protein AtI-2 physically interact with some SnRK2 family members.

To validate the interaction results obtained from the Y2H assays, we performed a split-luciferase complementation (Split-LUC) assay in tobacco leaves. As illustrated in Fig 1C, the co-expression of AtI-2-nLUC with cLUC-TOPP1 reconstituted luciferase activity, and served as our positive control. In contrast, co-expressing nLUC/cLUC, nLUC/cLUC-TOPP1, or AtI-2-nLUC/cLUC resulted in only background luciferase signals (Fig 1C). The co-expression of nLUC-tagged SnRK2.2, SnRK2.3 or SnRK2.6 with cLUC-TOPP1 produced detectable luciferase activity, confirming the results from the Y2H assay. Similarly, the co-expression of SnRK2.2-, SnRK2.3-, or SnRK2.6-nLUC with cLUC-AtI-2 led to luciferase activity that was comparable to another positive control, which co-expressed SnRK2.6-nLUC and ABI1-cLUC (Fig 1D). In addition, we performed in vitro pull down (S2 Fig) and co-immunoprecipitation assays in protoplasts, and the results support the interactions between SnRK2.6 and TOPP1 (Fig 1E) and between AtI-2 and SnRK2.6 (Fig 1F).

In mammals, INHIBITOR-2 (I-2) interacts with Protein Phosphatase 1 (PP1). I-2 promotes substrate recognition by PP1 and the formation of a trimeric I-2/PP1/substrate protein complex [29,30]. To investigate whether a similar trimeric Ati-2/TOPP1/SnRK2 complex might be possible, we co-expressed FLAG-tagged AtI-2 with cLUC-TOPP1 and SnRK2.6-nLUC in leaves. As shown in Fig 1G, the co-expression of SnRK2.6-nLUC with cLUC-TOPP1 generated a detectable but relatively weak luciferase signal. In contrast, the co-expression of FLAG-AtI-2, SnRK2.6-nLUC and cLUC-TOPP1 produced a much stronger luciferase signal. Western blot analysis confirmed that cLUC-TOPP1 and SnRK2.6-nLUC were expressed at comparable levels in the two samples (Fig 1G). These data suggest the possibility that AtI-2 may facilitate the association between TOPP1 and SnRK2s, and that the proteins might form a trimeric complex.

### TOPP1 and AtI-2 physically interact with some ABA receptors

**Fig 2:**
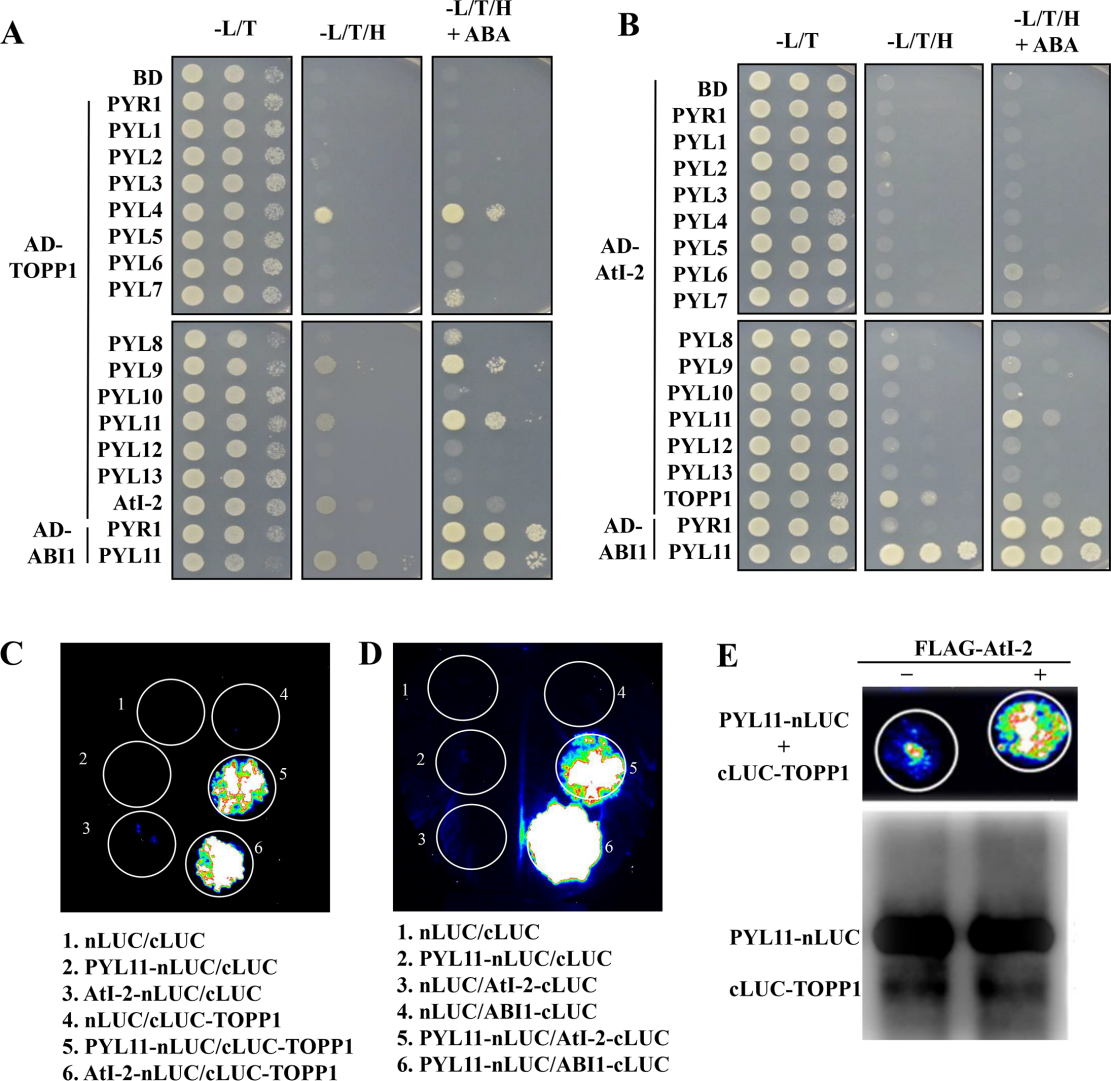
TOPP1 and AtI-2 physically interact with PYLs. TOPP1 interacts with PYLs in Y2H assay. AtI-2 interacts with PYLs in Y2H assay. TOPP1 or AtI-2 was fused to the GAL4-activating domain (AD) and PYLs were fused to the GAL4-DNA binding domain (BD). (A) TOPP1 interacts with PYL11 in split-LUC assay. (B) AtI-2 interacts with PYL11 in split-LUC assay. Alt-2 enhances the interaction between TOPP1 and PYL11 in split-LUC assay. Anti-LUC western blot shows the levels of the indicated fusion proteins. (C) The split-LUC assay were repeated at least three times with consistent results.

In the presence of ABA, the ABA receptors PYR1/PYLs associate with and inactivate the PP2Cs [23,31]. Since TOPP1 encodes a Ser-Thr phosphatase, we considered the possibility that TOPP1 might also interact with the ABA receptors. We performed Y2H assays to evaluate potential interactions between TOPP1 and PYLs. As shown in Fig 2A, yeast co-expressing AD-TOPP1 and BD-PYL4, -PYL9 or -PYL11 grew in the selection media in the absence of ABA. The exogenous application of ABA enhanced the interactions between TOPP1 and these PYLs (PYL4/9/11) (Fig 2A).

Since we found that both TOPP1 and AtI-2 interact with SnRK2s, we subsequently investigated whether, like TOPP1, AtI-2 may also interact with PYLs. Yeast co-transformed with AD-AtI-2 and BD-PYLs in yeast showed detectable growth on the SD-Leu/Trp/His medium, although the growth was less than yeast co-transformed with TOPP1 and PYLs, suggesting a weaker interaction (Fig 2B). Among fourteen PYLs, PYL11 showed the strongest interaction with AtI-2, and this interaction was largely dependent on ABA.

We validated some of these protein interactions with the Split-LUC assays. We tested PYL11 since it displayed stronger interactions with TOPP1 and AtI-2 in the Y2H assays. The nLUC and cLUC empty constructs expressed together or with individual fusion constructs resulted in only background luciferase activity. However, we found that co-expression of cLUC-TOPP1 with PYL11-nLUC produced a strong luciferase signal, comparable to the positive control of AtI-2-nLUC with cLUC-TOPP1 (Fig 2C), confirming that TOPP1 interacts with PYL11. Similarly, the co-expression of PYL11-nLUC with AtI-2-cLUC or the positive control ABI1-cLUC also exhibited strong luciferase activity (Fig 2D), verifying the interaction between AtI-2 and PYL11. To test whether AtI-2 may enhance the interaction between TOPP1 and PYL11, cLUC-TOPP1 and PYL11-nLUC were co-expressed with or without FLAG-AtI-2. As illustrated in Fig 2E, the luciferase signal generated by co-expression of cLUC-TOPP1 and PYL11-nLUC was notably increased by FLAG-AtI-2. Further western blot results showed equivalent protein levels of cLUC-TOPP1 and PYL11-nLUC. The data indicated that AtI-2 may also promote the interaction between TOPP1 and PYLs (Fig 2E).

### TOPP1 and AtI-2 suppress the kinase activity of SnRK2.6 *in vitro*

**Fig 3:**
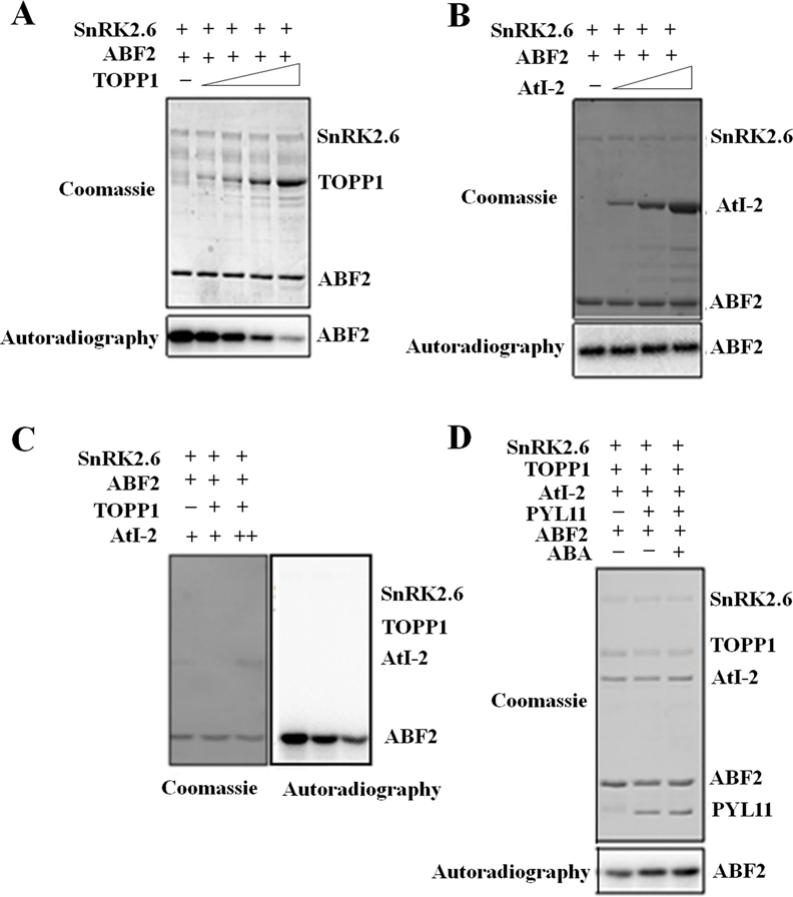
TOPP1 and AtI-2 inactivate SnRK2.6 *in vitro*. (A) TOPP1 diminishes SnRK2.6-catalyzed ABF2 phosphorylation. (B) AtI-2 does not affect SnRK2.6 activity. Recombinant protein MBP-SnRK2.6 and GST-ABF2 were incubated with GST-AtI-2. The phosphorylation of ABF2 was not reduced in the presence of AtI-2. (C) AtI-2 enhances the inactivation of SnRK2.6 by TOPP1. MBP-SnRK2.6 and GST-ABF2 were incubated with GST-TOPP1, or GST-AtI-2 or both GST-TOPP1 and GST-AtI-2. The SnRK2.6 activity was indicated by the radio-activities of GST-ABF2. (D) PYL11 partially prevents the inhibition of SnRK2.6 by TOPP1 or TOPP1/AtI-2. MBP-SnRK2.6 and GST-ABF2 were incubated with GST-TOPP1, or the combination of GST-TOPP1 and GST-AtI-2. GST-PYL11 was introduced with or without ABA. At least three independent experiments were performed in (A) to (D) with similar results.

TOPP1 encodes a Ser-Thr protein phosphatase in plants [32]. We purified GST-tagged TOPP1 (GST-TOPP1) and incubated it with the general substrate *p-*Nitrophenyl Phosphate (*p*NPP). As demonstrated in S3A Fig, TOPP1 displayed a phosphatase activity, which was reduced by the addition of AtI-2. Next, we tested whether TOPP1 affects SnRK2.6 activity. When TOPP1, SnRK2.6 and the SnRK2 substrate GST-ABF2 (Gly73 to Gln119) were incubated together, the SnRK2.6-mediated phosphorylation of ABF2 was reduced by TOPP1 in a dose-dependent manner (Fig 3A). Incubation of SnRK2.6 together with TOPP1 led to an inhibition of SnRK2.6 autophosphorylation (S3B Fig). Together, these data suggest that TOPP1 could dephosphorylate and inhibit the kinase activity of SnRK2.6 *in vitro*.

Since AtI-2 appeared to enhance the association of TOPP1 with SnRK2.6 in our Y2H and Split-LUC experiments, we sought to determine if AtI-2 also affects SnRK2.6 activity. We incubated recombinant SnRK2.6 with its substrate ABF2 and increasing amounts of GST-AtI-2. SnRK2.6-mediated ABF2 phosphorylation remained unaltered (Fig 3B), suggesting that AtI-2 does not affect SnRK2.6 activity by itself *in vitro*. However, AtI-2 enhanced the TOPP1-mediated reduction in ABF2 phosphorylation by SnRK2.6 (Fig 3C). The result also shows that SnRK2.6 does not phosphorylate AtI-2 *in vitro* (Fig 3C).

It has been known that PYLs bind to PP2Cs to inhibit their phosphatase activities. Thus, we wanted to test whether PYLs could also inhibit the phosphatase activity of TOPP1. We selected PYL11 as it showed the strongest interaction with TOPP1 and AtI-2 in our Y2H assay (Fig 2). As shown in S3C Fig, His-tagged PYL11 suppressed the phosphatase activity of TOPP1 in a dose-dependent manner *in vitro*.

We found that TOPP1 reduced SnRK2.6-mediated phosphorylation of ABF2, and that this reduction was enhanced by AtI-2 (Fig 3A and 3C). As PYL11 was shown to inhibit the phosphatase activity of TOPP1 *in vitro*, we further introduced PYL11 to examine the potential effect of PYL11 on the inhibitory activity of TOPP1-AtI-2 complex on SnRK2.6. The addition of PYL11 did not result in a substantial increase in SnRK2.6 phosphorylation of ABF2, although the phosphorylation appeared slightly enhanced in the presence of ABA (Fig 3D).

### TOPP1 and AtI-2 negatively regulate ABA signaling in protoplasts

**Fig 4:**
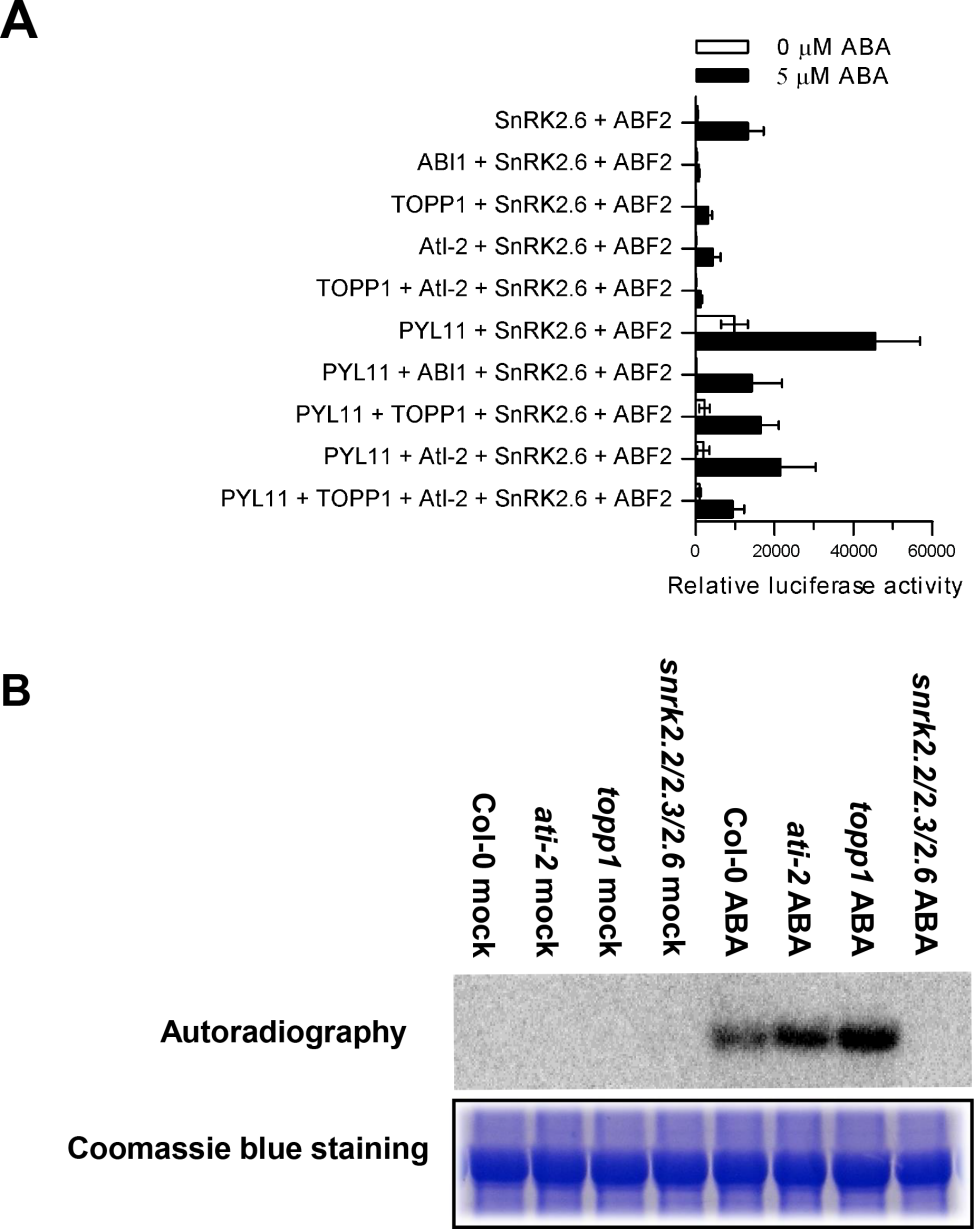
TOPP1 and AtI-2 suppress SnRK2.6 signaling. (A) TOPP1 and AtI-2 suppressed the activity of SnRK2.6, which can be released by PYL11 in the presence of ABA. ABA signaling pathway was reconstituted by co-expression of TOPP1, AtI-2, PYL11, SnRK2.6 and ABF2 in wild type (Col-0) protoplasts. The induction of *RD29B*::*LUC* was used as the ABA-responsive reporter, while *ZmUBQ*::*GUS* was used as the control for transformation efficiency. After co-transformation, protoplasts were incubated for 4 h under light without (close bars) or with 5 μM ABA (open bars). Relative luciferase activities were statistically summarized from at least three biological replicates. Error bars indicate SD. (*n* = 3). (B) Increased SnRK2.2/3/6 activities in *topp1* and *ati-2* mutants in in-gel kinase assay. The total proteins were extracted from 4-day-old seedlings without or with ABA treatment. Histone was used as a substrate for SnRK2.2/3/6. Coomassie blue staining indicates the equal loading. The experiment was repeated at least three times with similar results.

The results from the *in vitro* phosphorylation assays above suggest that TOPP1 and AtI2 may form a complex to suppress the kinase activity of SnRK2.6. To determine whether a TOPP1-AtI-2 complex functions as a negative regulator in ABA signal transduction, we performed transient expression assays in *Arabidopsis* mesophyll protoplasts. We employed a protoplast system in which ABA induces SnRK2.2/3/6-dependent ABF2 phosphorylation and, in turn, the expression of an ABA-responsive *LUC* reporter gene driven by the *RD29B* promoter (*RD29B-LUC*) [24]. We co-expressed TOPP1 and/or AtI-2 with SnRK2.2/3/6, ABF2 and *RD29B*-LUC in protoplasts with or without 5 μM ABA. GUS was also co-expressed to determine the transfection efficiency. As expected, we found that ABA induced *RD29B-LUC* expression in protoplasts co-expressing SnRK2.6 and ABF2, and the induction was suppressed by co-expressing ABI1 (Fig 4A). The additional co-expression of TOPP1 or AtI-2 with SnRK2.6 and ABF2 also suppressed ABA-induction of *RD29B-LUC*, indicating a negative role for TOPP1 and AtI-2 in ABA signaling (Fig 4A). Moreover, co-expression of both TOPP1 and AtI-2 with SnRK2.6 and ABF2 further suppressed *RD29B-LUC* induction by ABA. Together, these data suggest that TOPP1 and AtI-2 may function in a complex to suppress ABA-induced SnRK2.6 kinase activity (Fig 4A). Similar results were obtained for SnRK2.2 and SnRK2.3, indicating that SnRK2.2/2.3-dependent ABF2 phosphorylation and activation of *RD29B-LUC* was also inhibited by a TOPP1/AtI-2 complex (S4A and S4B Fig).

As reported previously [33], co-expressing PYL11 in the protoplasts released the suppression of *RD29B-LUC* expression by ABI1 (Fig 4A). Similarly, we found that PYL11 could release the inhibition of *RD29B-LUC* by TOPP1, AtI-2, and by the TOPP1-AtI-2 complex (Fig 4A), although it is difficult to tell whether the effect of PYL11 was due to inhibition of the co-transfected TOPP1 or due to inhibition of endogenous clade A PP2Cs. These protoplast transient expression results suggest that TOPP1 and AtI-2 collaborate to negatively regulate ABA signaling in a manner similar to the PP2Cs.

To acquire additional evidence that TOPP1 and AtI-2 negatively regulate SnRK2s *in vivo*, we performed an in-gel kinase assay to examine endogenous SnRK2 activity in WT, and T-DNA insertion mutants of *TOPP1* (*topp1*) and AtI-2 (*ati-2*). We isolated homozygous *topp1* and *ati-2* mutant plants and genomic DNA PCR confirmed that *topp1* and *ati-2* lines were homozygous as shown in S5A and S5B Fig. Previous studies have shown that the ABA-induced endogenous kinase activities of SnRK2s can be detected by in-gel kinase assays using histones as a substrate [13,34]. Our in-gel kinase assays revealed no detectable difference in the endogenous activities of SnRK2.2/3/6 in WT and mutant plants in the absence of ABA. However, upon ABA treatment for one hour, the phosphorylation signals corresponding to the endogenous kinase activities of SnRK2.2/3/6 were higher in *topp1* and *ati-2* mutant plants than those in the WT. As a control, no SnRK2.2/3/6 activities were detected in the *snrk2*.*2/2*.*3/2*.*6* triple mutant (Fig 4B). These data suggest that the TOPP1 and AtI-2 mutations resulted in increased kinase activities of SnRK2.2/3/6 in plants, further supporting their negative role in regulating ABA-induction of SnRK2.2/3/6 activities.

### *In planta* function of TOPP1 and AtI-2

**Fig 5:**
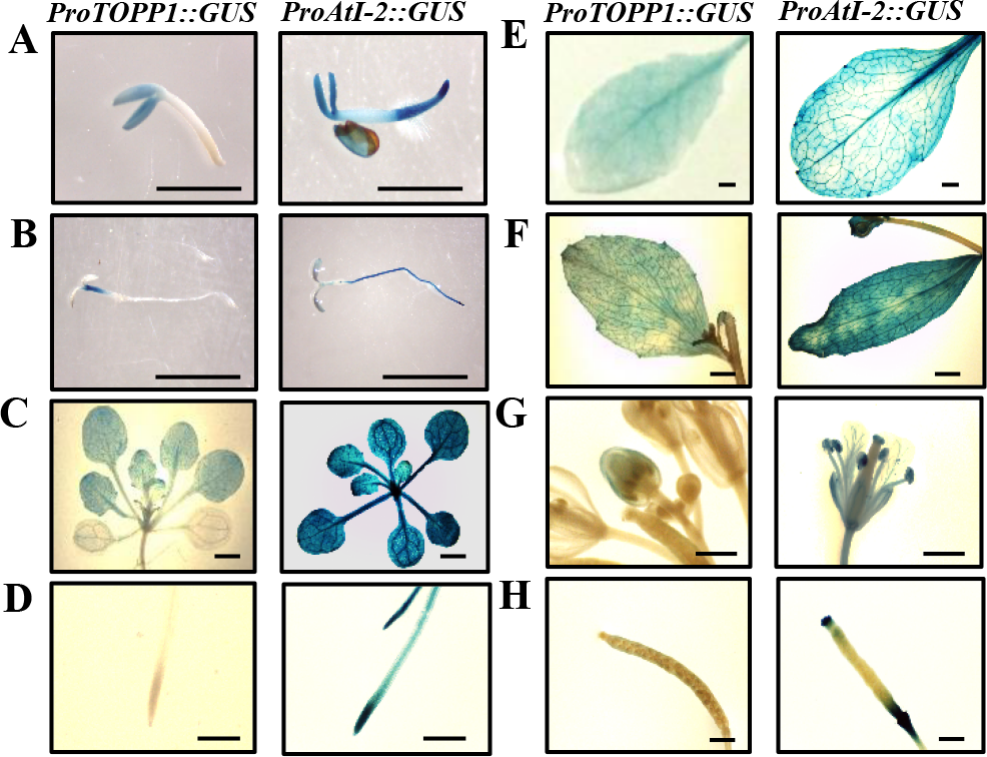
Expression patterns of *TOPP1* and *AtI-2* as indicated by *promoter*::*GUS* activities. (A) to (H) GUS staining of transgenic plants expressing *ProTOPP1*::*GUS* and *ProAtI-2*:*GUS* at different growth stages. Typical staining images of germinating (A) and 4-day-old (B) seedlings, aerial tissues (C) and roots from 2-week-old seedlings (D), rosette leaves (E) and cauline leaves (F) and flowers (G) as well as siliques (H) from 6-week-old plants are shown. Scale bar = 1 mm.

We generated transgenic lines with the promoters of *TOPP1* or *AtI-2* driving a β-glucuronidase (*GUS*) reporter to help determine the expression patterns of *TOPP1* and *AtI-2* in plants. Seeds of transgenic plants expressing *ProTOPP1*:: *GUS* and *ProAtI-2*:: *GUS* were first germinated on MS plates and then transferred to soil to examine *GUS* expression at different developmental stages. GUS staining of germinating seeds and 4-day-old seedlings showed that *ProTOPP1*:: *GUS* is expressed mainly in the cotyledon, while *ProAtI-2*: *GUS* is expressed throughout the whole seedling, with some enrichment in the roots (Fig 5A and 5B). In two-week-old seedlings, GUS activity was detected in all true leaves and roots but not in the cotyledons in *ProTOPP1*:: *GUS* transgenic lines, whereas *ProAtI-2*: *GUS* was strongly expressed in all tissues (Fig 5C and 5D). In adult plants, GUS activity was detected in rosette and cauline leaves but not in the stems (Fig 5E and 5F). *ProAtI-2*::*GUS* transgenic plants displayed higher GUS activity in the flower and silique than *ProTOPP1*:*GUS* transgenic plants (Fig 5G and 5H). *ProTOPP1*:: *GUS* expression was much weaker overall compared to that of *ProAtI-2*: *GUS*, possibly because that some regulatory sequences may be missing from the *TOPP1* promoter in the construct (Fig 5), which makes it difficult to conclude whether *TOPP1* is not expressed in certain tissues.

**Fig 6:**
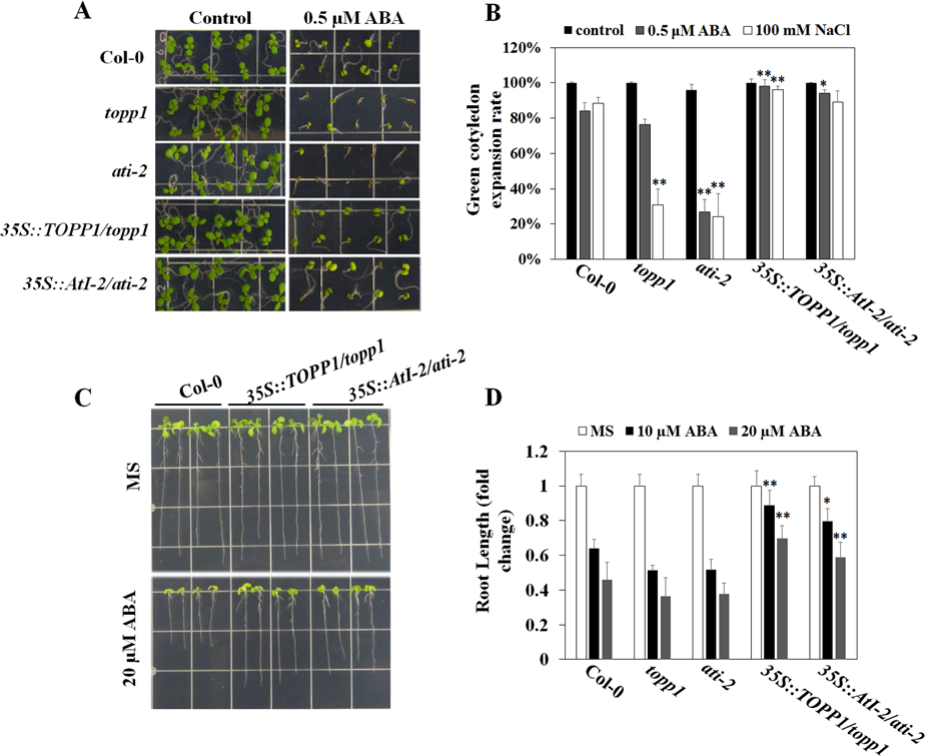
Effect of TOPP1 and AtI-2 on ABA and salt sensitivity. (A) ABA sensitivity of *topp1* and *ati-2* mutants as well as the *TOPP1* and *AtI-2* over-expression lines during germination and early seedling growth. (B) Quantification of the green cotyledon expansion rates of indicated genotypes in response to ABA and NaCl. The green cotyledon expansion rate of indicated genotypes was recorded at 7 days after germination. At least 50 seeds per genotype were sterilized and germinated on ½ MS agar plates with or without 0.5 μM ABA or 100 mM NaCl in each replicate. Seedlings with green cotyledon expansion were counted. Data indicate mean values± SD from three replicates. Significance between the mean values were analyzed with Student’s *t* test (* P˂ 0.05). (C) The ABA-mediated inhibition of seedling growth of wild type, and *TOPP1* and *AtI-2* over-expression lines. Photographs were taken at 7 days after seedlings transfer to ½ MS plates, 10 μM ABA or 20 μM ABA containing MS plates. At least ten 3-day-old seedlings from indicated genotypes were transferred and root length was recorded after 7 days. The experiments were repeated at least three times with similar results. (D) Quantification of the fold changes of primary root length of wild type, *topp1*, *ati-2*, and *TOPP1* and *AtI-2* over-expression lines at 7 days after transfer. Values are means ± SD (*n* = 30). Significance between the mean values were analyzed with Student’s *t* test (* P˂ 0.05, ** P˂ 0.01).

To help understand the potential function of TOPP1 and AtI-2 in regulating ABA and stress responses in plants, we generated *TOPP1* and *AtI-2* transgenic lines by transforming *topp1* or *ati-2* mutant plants (S5A and S5B Fig) with *35Spro*::*TOPP1-HA* and *35Spro*::*AtI-2-FLAG*, respectively. qRT-PCR assays showed that *TOPP1* and *AtI-2* are not expressed in their respective mutants but are highly expressed in the transgenic lines (S5C Fig). When germinated on MS plates with 0.5 μM ABA, the *topp1* and *ati-2* mutations did not affect seed germination, but the *topp1* mutant plants showed a slightly reduced rate of green cotyledon expansion compared with wild type plants while *ati-2* displayed a significantly lower rate of green cotyledon expansion (Fig 6A and 6B). The expression of *35Spro*::*TOPP1-HA* and *35Spro*::*AtI-2-FLAG* rescued the post-germination hypersensitivity phenotypes of the loss-of-function mutants; the green cotyledon expansion rates in the transgenic plants were slightly higher than that in the WT (Fig 6A and 6B). We also examined post-germination root growth. Although the root length of *topp1* or *ati-2* mutants was indistinguishable from WT (S6 Fig), we found that the roots of the transgenic lines expressing *35Spro*::*TOPP1-HA* and *35Spro*::*AtI-2-FLAG* were significantly longer than WT roots after growing on ABA-containing MS plates, thus revealing an increased tolerance to exogenous ABA (Fig 6C and 6D).

We also tested whether *topp1* and *ati-2* mutant plants may have altered sensitivity to salt stress. Similar to their ABA-hypersensitive seed germination phenotype, both *topp1* and *ati-2* exhibited increased sensitivities to salt stress, with severely retarded germination and barely any green cotyledon expansion in the presence of 100 mM NaCl. Consistently, the expression of *35Spro*::*TOPP1-HA* and *35Spro*::*AtI-2-*FLAG suppressed the hypersensitivity of the loss-of-function mutants (Figs 6B and S7). Moreover, water loss rate from detached leaves was reduced in *ati-2* compared with WT plants while *topp1* showed an overall rate of water loss that was similar to the WT although the rate appeared to be unsteady (S8A Fig). In contrast, the *35Spro*::*TOPP1-HA* expression lines showed an increased water loss compared to WT whereas the *35Spro*::*AtI-2-*FLAG expression lines were indistinguishable from WT (S8B Fig). Together, the genetic evidence suggests that TOPP1 and AtI-2 negatively regulate ABA sensitivity during seed germination and early seedling growth.

### TOPP1 and AtI-2 negatively regulate ABA-responsive gene expression in plants

**Fig 7:**
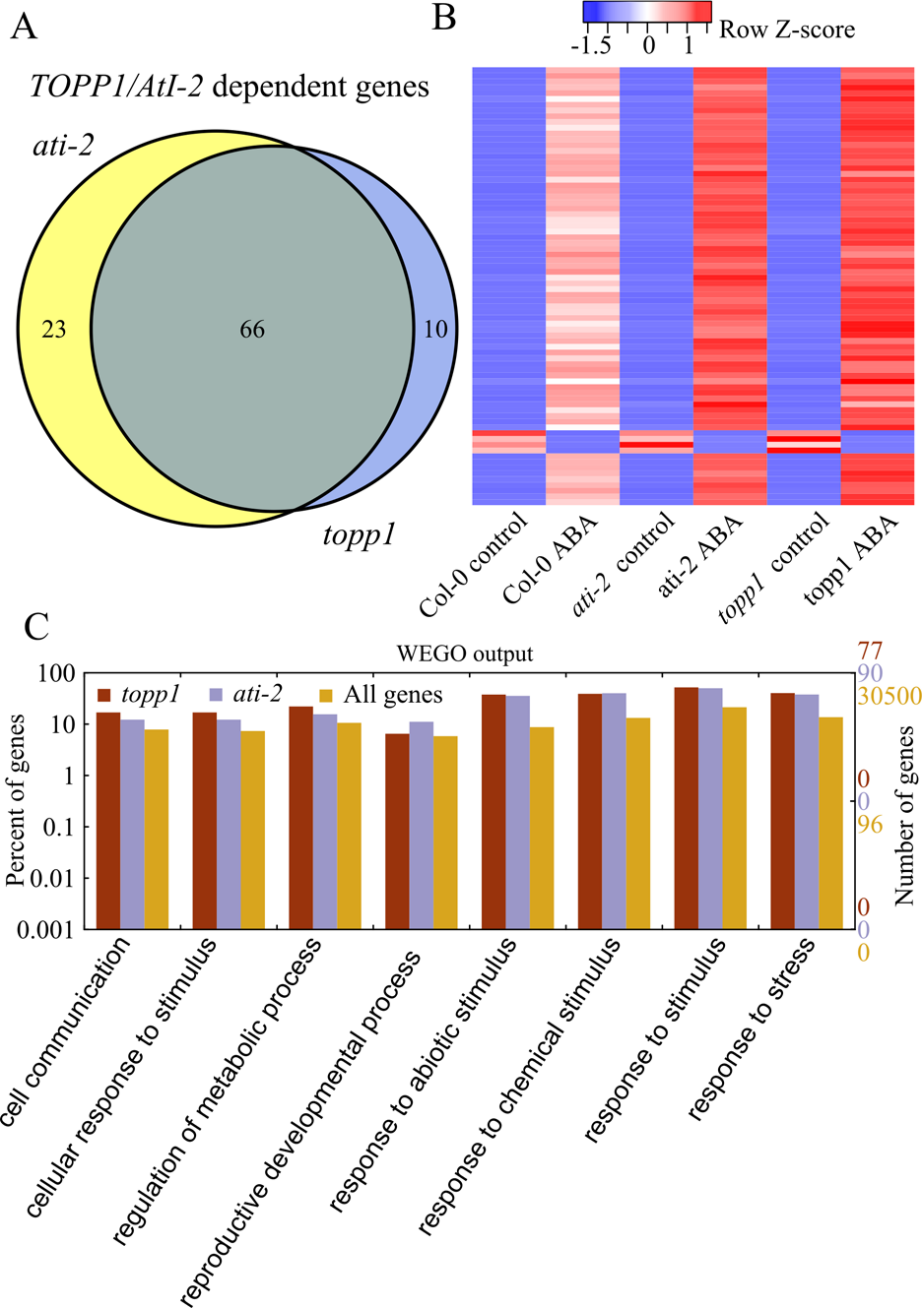
RNA-seq analysis shows the overlapping pattern of transcriptome changes between *topp1* and *ati-2* in response to ABA. (A) The Venn diagram represents the number of genes that were significantly changed at least 1.2-fold in *topp1* or *ati-2* mutant plants than wild-type after ABA treatment (FDR value < 0.05; fold change ≥1.2). Differentially expressed ABA responsive genes were identified by DESeq (P-value < 0.05; fold change > 4 after ABA treatment). (B) Heatmap depiction of the ABA-responsive gene expressions in wild type, *topp1* and *ati-2*. Heatmap was created by heatmap.2 function of gplots package in R. (C) Gene ontology enrichment analysis of the differentially expressed (DE) genes co-regulated by *TOPP1* and *AtI-2*. The statistically enriched GO categories were shown based on the number and percentage of DE genes (p value < 0.05).

To investigate the genome-wide effect of TOPP1 and AtI-2 on ABA-induced gene expression changes, we performed RNA-sequencing experiments. In total, 76 and 89 genes displayed at least 1.2-fold changes in expression level in *topp1* and *ati-2* mutant plants relative to WT plants upon ABA treatment (Fig 7A). Of these, 66 genes were common in both mutant plants, indicating that they were co-regulated by TOPP1 and AtI-2. Interestingly, several ABA-responsive genes such as *RESPONSIVE TO DESSICATION 29A* (*RD29A*), *COLD-REGULATED 15A* (*COR15A*) and *COR47* were in the list of differentially expressed (DE) genes co-regulated by TOPP1 and AtI-2 (S1 Dataset). The heat map generated with the DE genes revealed a highly similar pattern between *topp1* and *ati-2* (Fig 7B). Gene ontology analysis indicated that the DE genes co-regulated by TOPP1 and AtI-2 were enriched in stress-related biological processes (Fig 7C), consistent with TOPP1 and AtI-2 functioning in ABA and salt stress responses.

**Fig 8:**
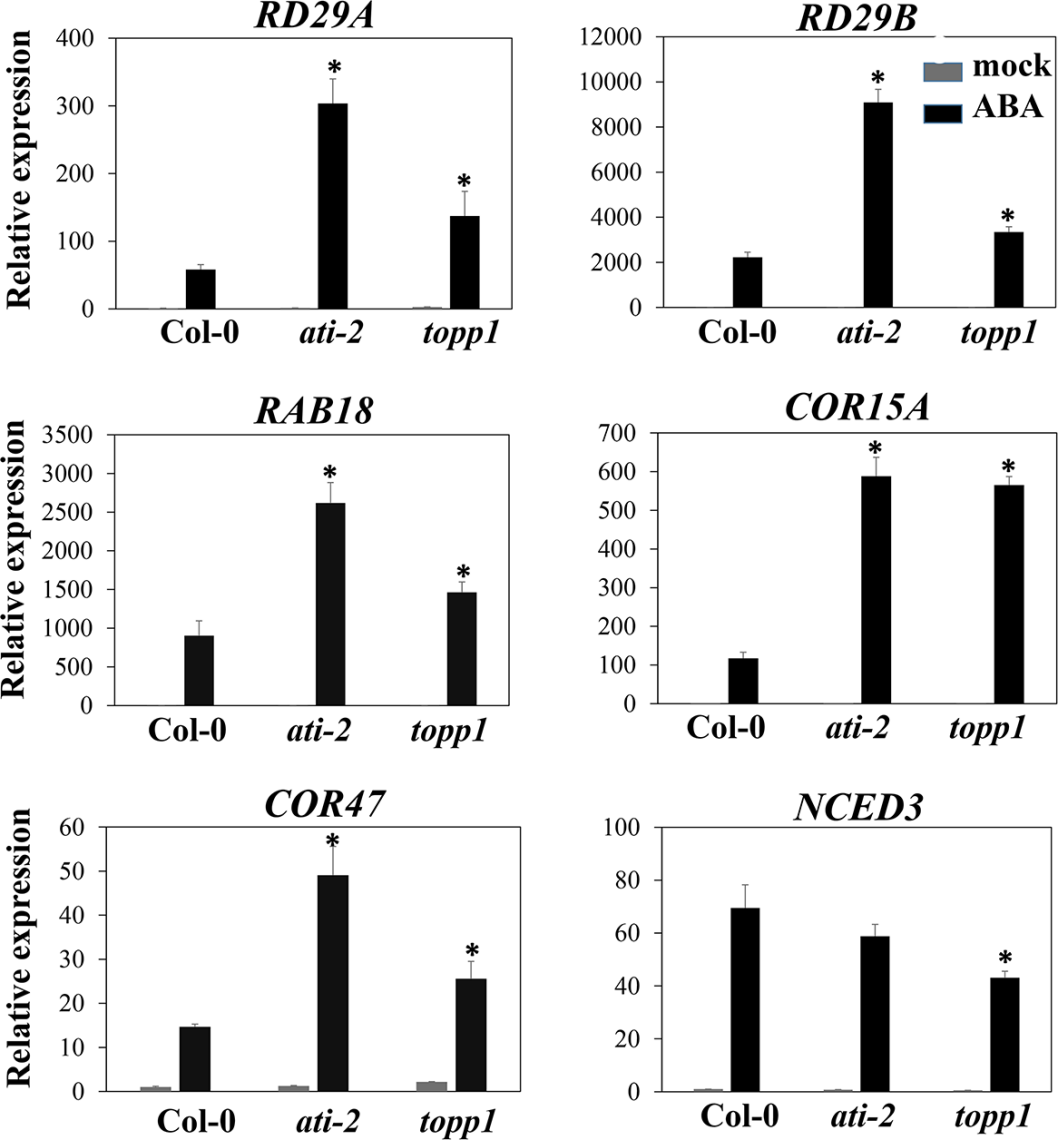
Increased expression of ABA-responsive genes in *ati-2* and *topp1* mutants in response to ABA. Ten-day-old seedlings were treated with or without 50 μM ABA for 3 hours. Relative transcript levels were normalized against the reference gene *ACTIN2*. The data represent mean value ± SD from three biological replicates. Asterisk indicates significant differences (P≤0.05).

To validate the results from RNA-seq, we examined the expression of several ABA-responsive genes including *RD29A*, *RD29B*, *RESPONSIVE TO ABA 18* (*RAB18*), *COR15A* and *COR47* in the presence or absence of 50 μM ABA for 3 hours by quantitative Real-Time PCR (RT-qPCR). In the presence of ABA, the expression of these genes was substantially higher in both *ati-2* and *topp1* mutant plants compared to WT plants (Fig 8). In contrast, the expression of *NINE-CIS-EPOXYCAROTENOID DIOXYGENASE 3* (*NCED3*), which encodes a key enzyme in ABA biosynthesis, was induced less by ABA in *topp1* mutant plants relative to WT (Fig 8). These results further support the conclusion that AtI-2 and TOPP1 negatively regulate ABA signaling.

## Discussion

**Fig 9:**
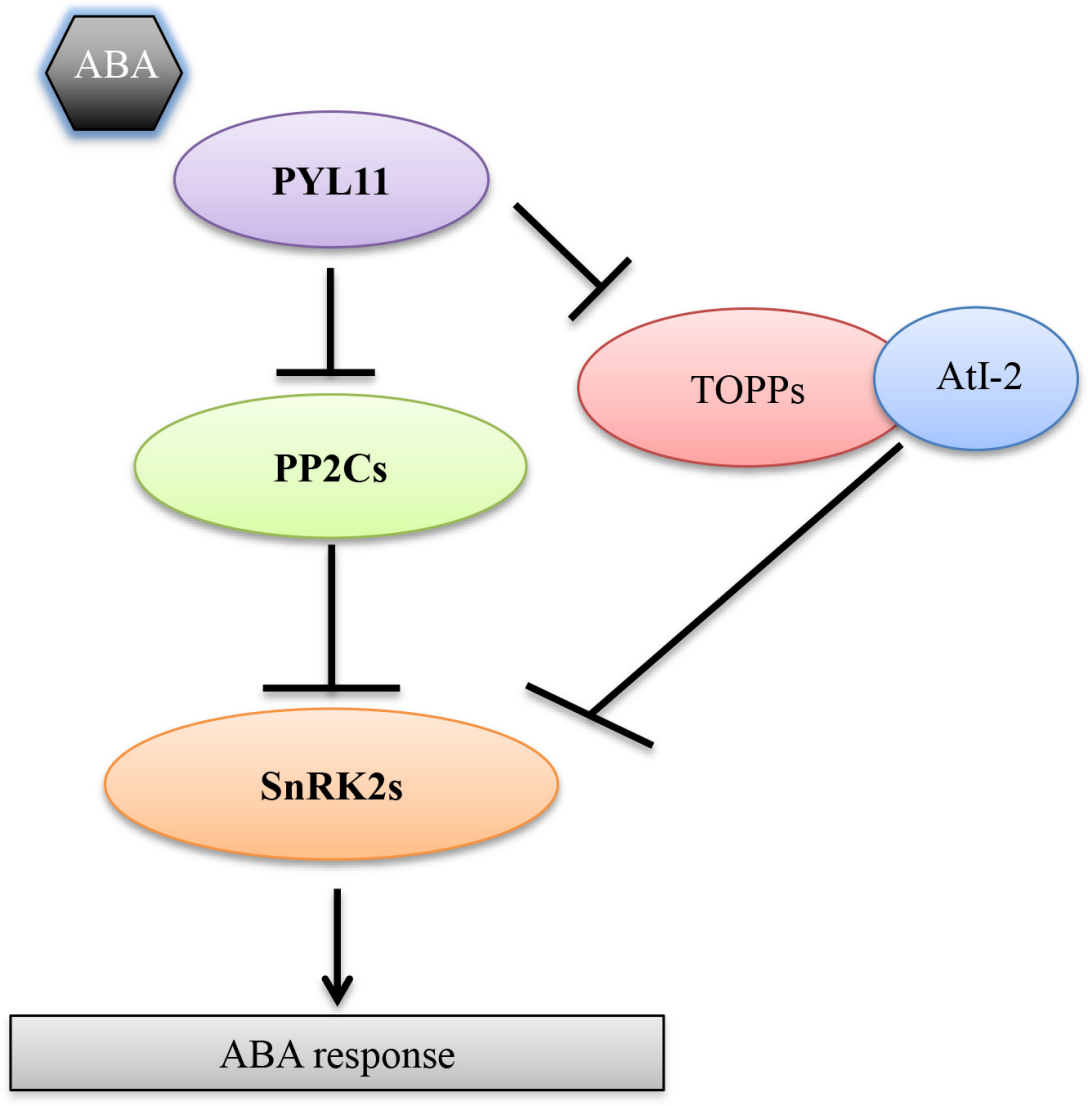
A proposed model of TOPP1- and AtI-2-mediated ABA signaling pathway. TOPP1-AtI-2 complex can modulate ABA signaling through physical interactions with PYL11 and SnRK2s. Binding of TOPP1 and AtI-2 with SnRK2.6 can suppress the kinase activity of SnRK2.6. Such suppression can be released at least partially by PYL11 in the presence of ABA.

Although substantial progress has been made in our understanding of ABA perception and signal transduction, the complexity of the signaling pathway remains to be fully explored. In the present study, we have provided new insights into ABA signaling by identifying two new components, TOPP1 and its regulatory protein AtI-2. Our results suggest that TOPP1 and AtI-2 form a complex and negatively regulate ABA signaling through a physical interaction with PYLs and SnRK2s in plants. TOPP1 and AtI-2 function together to inhibit SnRK2s, but the inhibition appears to be partially released by PYL11. Therefore, we propose that TOPP1 and possibly TOPP1 paralogs function together with AtI-2 and act analogously to the PP2Cs (Fig 9), but due to their different biochemical and expression features from the PP2Cs, they may confer additional specificity and flexibility to ABA signaling.

TOPP1 belongs to the serine/threonine phosphatase family (PSPs), which is composed of three major groups, including phosphoprotein phosphatases (PPPs), metal-dependent protein phosphatases (represented as PP2Cs), and the aspartate-based phosphatases [35]. Representative subgroups of the PPP family can be further categorized as PP1, PP2A, PP2B, PP4, PP5, PP6, and PP7 [35]. PP1 is a widely expressed catalytic subunit across all eukaryotes, with a Mg^2+^/ Mn^2+^-dependent activity *in vitro* [36]. Unlike PP2Cs, which contain both catalytic and regulatory domains within the same polypeptide chain, PP1 is tightly controlled by its regulatory proteins that affect its substrate specificity and catalytic activity [36]. In mammals, PP1 is known to be involved in controlling various cellular processes such as glycogen metabolism, cell division and RNA splicing [37,38]. In *Arabidopsis*, PP1 is annotated as TOPPs, with nine members ubiquitously expressed in most tissues [36,39]. Previous studies have shown that ABA-invoked PYLs bind and inhibit PP2Cs [40]. We found that TOPP1 interacts with some PYLs. The TOPP1-PYL interactions were ABA-independent, but could be enhanced by ABA. In comparison with the PP2Cs, TOPP1 appeared to display different patterns of pairwise interaction with PYLs. Since some PYLs have been reported to function preferentially under certain abiotic stress conditions [41,42], it is possible that TOPP1 functions with certain PYLs to transduce a tissue- or stress-specific signal.

Although numerous kinases in plants are well studied, only a few phosphatases have been characterized thus far. There are more than 1050 kinases reported in the *Arabidopsis* genome, whereas less than 150 annotated catalytic subunits of phosphatases have been reported [43]. Therefore, PP1 may mediate multiple cellular responses by targeting different kinases. Here, we provided biochemical evidence for TOPP1-mediated inhibition of SnRK2s *in vitro* and *in vivo*. Consistently, the endogenous SnRK2.2/2.3/2.6 activities in *topp1* mutant were higher than those in WT plants after ABA treatment, further supporting the negative roles of TOPP1and AtI-2 in the ABA signaling pathway.

To date, *Arabidopsis* Inhibitor-2 (AtI-2), Inhibitor-3 (I-3) and PP1 regulatory subunit 2-like protein 1 (PRSL1) have been characterized as regulatory proteins of TOPPs in *Arabidopsis* [44,45]. AtI-2 is known to interact with TOPPs through three conserved motifs identified by bioinformatics analysis. Biochemical studies of AtI-2 revealed its role in inhibiting the phosphatase activities of TOPPs1-9 *in vitro* [46]. It has been shown that TOPPs co-localize with AtI-2 in both the cytosol and nucleus, with an enriched signal in the nucleus [46,47]. Recently, it was reported that TOPP1 along with AtI-2 participated in stomatal opening downstream of the blue-light sensing kinase phototropin and upstream of the H^+^-ATPase [47,48].

Our results suggest that AtI-2 could function beyond inhibition of TOPPs in plants. Our spilt-LUC assays indicated that an enhancement by AtI-2 on the interactions between SnRK2.6 and TOPP1 or PYL11. These results indicate the possibility that AtI-2 may stabilize the interaction of TOPP1 with its targets. Although AtI-2 did not suppress SnRK2.6 activity alone *in vitro*, it was able to enhance the inactivation of SnRK2.6 by TOPP1, which was probably achieved by facilitating the TOPP1-SnRK2.6 interaction. On the contrary, the transactivation data in protoplasts revealed that AtI-2 alone could suppress the SnRK2.2/3/6-ABF2 mediated *RD29B*-*LUC* expressions, which is not fully consistent with the results obtained from *in vitro* kinase assay. One explanation for this discrepancy would be that AtI-2 could promote the activities of endogenous TOPPs in the protoplasts. Alternatively, AtI-2 could also suppress SnRK2s activities by modulating other phosphatases or undefined factors. In addition, gene expression analyses showed an overlapping function of TOPP1 and AtI-2, as they co-regulated various groups of genes in response to ABA. Together, the *in vitro* and *in vivo* data provide strong evidence for the coordinated inhibition of ABA signaling by AtI-2-TOPP1.

In summary, we have provided new insights into ABA signaling by characterizing the roles of two new components, TOPP1 and AtI-2, in the pathway. AtI-2 promotes TOPP1 suppression of SnRK2s, thus negatively regulating ABA signaling. Our results suggest that the TOPP1-AtI-2 complex helps to fine-tune the core signaling pathway mediated by PP2Cs.

## Material and Methods

### Plant materials and growth conditions

*Arabidopsis thaliana* Columbia-0 ecotype (Col-0) was used in this study. T-DNA insertion mutants, *topp1* (SALK_057537), and *ati-2* (SALK_110571C) were obtained from the Arabidopsis Biological Resource Center (ABRC, Columbus, OH). Homozygous mutants were isolated by genomic PCR. (Primer sequences are listed in S2 Dataset). Double mutant *snrk2*.*2/2*.*3* was obtained as described in Fujii *et al*, 2007 [15]. *Arabidopsis* seedlings were grown on horizontal Murashige and Skoog (MS) medium containing full MS salts, 3% (w/v) sucrose, and 0.6% (w/v) agar, pH 5.7 in growth chamber at 23°C under long day photoperiod condition (16 h light/8 h dark). Root growth inhibition assays and ABA- or salt-mediated seed germination and were performed as described previously (Fujii *et al*, 2009). The seeds were harvested at the same time and were used for the germination, cotyledon green expansion and post-germination root growth assays. For post-germination root growth assays, 3-days-old seedlings were first germinated on vertical MS medium and then were transferred to ABA-supplemented medium (10 μM) and the primary root growth was measured at 7 days after transfer. *Nicotiana benthamiana* was grown in growth room under 16 h light/8 h dark. One-month-old tobacco plants were used for transient expression assays.

### Y2H assays

The full-length coding sequences of *SnRK2s* and *PYLs* were PCR amplified using Phusion high fidelity Taq polymerase (New England Biolabs), cloned into the pGBKT7 vector (Gal4 DNA binding domain; Clontech), and used to screen a cDNA library for interacting proteins. An *Arabidopsis* cDNA library was prepared by Clontech in the pGADT7-RecAB vector (Gal4 activation domain; Clontech). Primers used for the Y2H assay were listed in S2 Dataset.

To confirm the protein interactions, pGADT7 plasmids containing TOPP1 or AtI-2 were co-transformed with members of pGBKT7-SnRK2s or pBDGal4-PYLs into *Saccharomyces cerevisiae* AH109 cells according to the standard yeast PEG transformation method [49]. Successfully transformed colonies were identified on yeast SD medium lacking Leu and Trp. To verify the protein interactions, colonies were transferred to selective SD medium lacking Leu, Trp, His in the absence or presence of 10 μM ABA. 1 mM 3-amino-1, 2, 4-triazole (3-AT) was added to reduce the self-activation effect of BD-SnRK2s. To determine the intensity of protein interaction, saturated yeast cultures were diluted to 10^−1^, 10^−2^ and 10^−3^ and spotted onto selection medium. Photographs were taken after 4 days incubation at 30°C.

### Generation of transgenic plants

To make *proTOPP1*:*GUS* and *proAtI-2*:*GUS* constructs, DNA fragments covering roughly 2 kb upstream to the translational initiation start codon sites were amplified by PCR from Col-0 genomic DNA as templates, the PCR products were then inserted into pENTR/D-TOPO vectors according to the manual (Invitrogen). Those promoter fragments were next transferred to destination vector pMDC162 by LR reaction with Gateway LR Clonase II enzyme mix (Invitrogen).

To generate overexpression lines, the coding sequences of *TOPP1* and *AtI-2* were amplified from Col-0 cDNA and then cloned into pENTR/D-TOPO vectors. The coding sequences were subsequently transferred to destination vectors to generate CaMV 35S promoter driven TOPP1 (pEarley201-TOPP1) and AtI-2 (pEarley202-AtI-2). After sequencing, those destination constructs were transformed into *Agrobacterium tumefaciens* GV3101 and were transformed into their mutants by the floral dipping method [50]. Transformed T0 seeds were harvested and were selected by Hygromycin B or Basta. Resistant T1 seedlings were grown in soil to obtain T2 generation. Homozygous T3 plants were used for phenotyping or GUS staining.

### Split luciferase (LUC) complementation assay

The coding sequences of proteins described in our work were amplified by PCR using primers listed in Supplement data set 2. The PCR products were first cloned into pENTER vector (Invitrogen) and then transferred to nLUC/cLUC vectors via LR reactions. Split-LUC complementation assay was performed by transient expression in leaves of *N*. *benthamiana* by agrobacterium-mediated infiltration. Briefly, *Agrobacterium* GV3101 strains carrying cLUC and nLUC constructs were cultured at 28°C overnight and then were re-suspended in the injection buffer (10mM MgCl_2_, 10mM MES and 100 μM Acetosyringone, pH 5.6) and incubated at room temperature for at least 3 h. Corresponding cLUC and nLUC constructs were equally mixed (final concentration OD_600_ = 0.5/each) and infiltrated into leaves of *N*. *benthamiana*. The infiltrated leaves were covered with plastic lid to maintain high humidity. After 2 days infiltration, leaves co-expressing different constructs were then examined for LUC activity by applying the LUC substrate D-luciferin (Promega) with CCD camera equipped with Winview software (Princeton instruments). The expressions of n/cLUC fusion proteins were determined by western blot with poly anti-Luciferase antibody (Sigma).

### Expression and purification of recombinant proteins from *E*. *coli*

The coding sequences of TOPP1 and AtI-2 were amplified by PCR and were cloned into pGEX4T1 vector (Amersham) with *EcoR*I/*Xho*I (TOPP1) and *BamH*I/*Xho*I (AtI-2), respectively. Recombinant proteins were purified using glutathione–agarose beads (GST) (Sigma-Aldrich). PYL11 was cloned into pET28a vector (Novagen), and MBP-SnRK2.6 was prepared as reported in Fujii. *et al* 2009. *Escherichia coli* (*E*. *coli*) BL21 (DE3) strains carrying pET28a-PYL11 and pMal-c2X-SnRK2.6 constructs were purified by Ni-NTA agarose (QIAGEN) and amylose resin (NEB), respectively as described by manufacturer’s protocol. pGEX4T1-ABF2 (Gly-73 to Gln-119) was prepared as described in Fujii. *et al* 2007. Primers used in this study are listed in S2 Dataset.

### In vitro pull down assay

Recombinant proteins were expressed and purified from *E*. *coli* according to the manufacturer’s protocol. Prey proteins of interest were incubated with the immobilized bait protein for 4 hours at 4°C. After binding *in vitro*, unbound prey proteins were washed with 1XPBS buffer for 4 times and the eluted proteins were boiled in 2XSDS loading dye. The protein samples were then separated by SDS-PAGE.

### Co-immunoprecipitation

The co-IP assays were performed in Arabidopsis protoplasts as described [51]. Full length of SnRK2.6 was amplified and cloned into pHBT95 with MYC tag through transfer PCR. The indicated plasmids were isolated using Maxi Prep kits (Qiagen). Briefly, After co-transformation, protoplasts were collected and suspended in 2 mL lysis buffer (50 mM Tris-HCl, pH 7.4, 150 mM NaCl, 1 mM EDTA, 1 mM DDT, 0.1% (v/v) Triton X-100, and 1× protease inhibitor cocktail from Sigma plus 1 mM PMSF) in ice for 15 min and then centrifuged at 12000 rpm for 10 min at 4°C. The supernatant was incubated with pre-balanced 20 μL monoclonal anti-HA-agarose antibody (Sigma) or anti-MYC-agarose (Abcam) at 4°C for at least 4 h with gentle rotation. The beads were washed at least four times with lysis buffer and boiled in 50 μL of 1×SDS loading buffer for 5 min. Samples were subjected to Western blots and detected with indicated antibodies.

### p-nitrophenyl phosphate (pNPP) phosphatase activity assay

General substrate p-nitrophenyl phosphate (pNPP, Sigma) was used to measure the phosphatase activity of TOPP1. Reactions were performed in assay buffer containing 50 mM Tris-HCl, pH 7.5, 2 mM MnCl_2_, 1 mM EDTA, 0.5% β-mercaptoethanol, 2 mg/ml BSA and 50 mM pNPP. An increasing gradient of recombinant protein GST-AtI-2 or His-PYL11 was incubated with GST-TOPP1 at 37°C for 1 h. After incubation, the reactions were quenched with 5 volumes of 0.5 M EDTA. The hydrolysis of pNPP was measured by following the absorbance at 405 nM (A_405_).

### *In vitro* kinase assay

The purified recombinant proteins GST-TOPP1, GST-AtI-2 and MBP-SnRK2.6 were incubated at room temperature for 20 min in kinase reaction buffer (25 mM Tris-HCl pH 7.4, 12 mM MnCl_2_, 1 mM DTT). After pre-incubation, GST-ABF2 and 1 μCi [ϒ-^32^P] ATP were added and incubated at 30°C for another 15min. After the reaction, 4X SDS-PAGE sample buffer was added to the reaction mixture and boiled for 5 min, the samples were separated by SDS-PAGE. Radioactivities of MBP-SnRK2.6 and GST-ABF2 were detected by the phosphoimager (BIO-RAD).

### In gel kinase assay

This assay was performed as described in Wang *et al* 2010. Briefly, seeds were germinated in liquid MS medium with 1.5% sucrose for 4 days and then were transferred to control MS medium or MS medium containing 50 μM ABA for 1 h. Total protein was extracted in lysis buffer containing 5 mM EDTA, 5 mM EGTA, 2 mM DTT, 10 mM NaF, 10 mM Na_3_VO_4_, 50 mM β-glycerophosphate, 5% glycerol, 1 mM phenylmethylsulfonyl fluoride (PMSF), 5 μg/mL leupeptin, 5 μg/mL aprotinin, and 100 mM HEPES-KOH, pH 7.5. After centrifugation at 14,000 rpm for 20 min, protein concentrations of supernatants were determined using Bradford reagent (BIO-RAD) with BSA as standard [52]. Total proteins (15 μg/lane) were separated in a 10% SDS-PAGE gel containing 2 mg/mL histone as a substrate. The gel was washed for 30 min three times with washing buffer (0.5 mM DTT, 5 mM NaF, 0.1 mM Na_3_VO_4_, 0.5 mg/mL BSA, 0.1% Triton X-100, and 25 mM Tris-HCl, pH 7.5), and then incubated for 1 h at room temperature with renatured buffer (1 mM DTT, 5 mM NaF, 0.1 mM Na_3_VO_4_, and 25 mM Tris-HCl, pH 7.5), and at 4°C overnight. After 30 min of incubation in reaction solution (2 mM EGTA, 12 mM MgCl_2_, 1 mM DTT, 0.1 mM Na_3_VO_4_, and 25 mM Tris-HCl, pH 7.5) at room temperature, the gel was incubated in 30 mL of reaction solution supplemented with 50 μCi of [ϒ-^32^P]ATP and 200 nM cold ATP for 60 min at room temperature. The gel was washed with 5% TCA and 1% sodium pyrophosphate for total 6 h with five time changes of buffer. The gel was dried and exposed to phosphoscreen overnight.

### Protoplast preparation and transient expression assay

Protoplast transient assays were performed as described (Fujii *et al* 2009). All the plasmids used in this assay were purified using QIAGEN Plasmid Maxi or Midi Kit. Briefly, plants were grown under 8 h light/16 h dark photoperiod condition. Leaf strips were incubated in enzyme solution (20 mM MES, pH 5.7, 1.5% (w/v) cellulase R10, 0.4% (w/v) macerozyme R10 (Yakult Pharmaceutical Industry), 0.4 M mannitol, 20 mM KCl, 10 mM CaCl_2_, 1 mM 2-mercaptoethanol and 0.1% BSA) for vacuum infiltration for 30 min and then were incubated for another 3 h at room temperature under the dark condition. Protoplasts was next filtered with a 75 μm nylon mesh and centrifuged at 100 g for 2 min in a 30 mL round bottomed tube, pellets were resuspended in W5 solution gently and precipitated at room temperature for 30 min.

Before transformation, protoplasts were replaced with MMg solution (4 mM MES, pH 5.7, 0.4 M mannitol and 15 mM MgCl_2_) to a final concentration of 2×10^5^ cell/ml. Protoplasts (200 μL) were mixed with indicated plasmids and 220 μL PEG solution (40% w/v PEG-4000, 0.2 M mannitol, and 100 mM CaCl_2_) and then mixed thoroughly. Protoplast and plasmids were incubated for 5 min, and washed with 440 μl of W5 solution; later pellets were resuspended in 50 μL of WI solution (4 mM MES, pH 5.7, 0.5 M mannitol and 20 mM KCl). After transfection, protoplasts were incubated in WI solution without or with 5 μM ABA under light, and protoplasts were harvested after 4 h incubation, then frozen in liquid N_2_ and stored at -80°C.

The frozen protoplasts were resuspended in 50 μL lysis buffer (2.5 mM Tris-phosphate, pH 7.8, 1 mM DTT, 2 mM DACTAA, 10% (v/v) glycerol and 1% (v/v) Triton X-100), and 20 μL of protoplast lysates were mixed with 100 μL of D-luciferin mix (Promega) for the measurement of LUC luminescence intensity with Wallac VICTOR2 plate reader (Perkin Elmer). Another 2 μL protoplast lysates were mixed with 10 μL of 4-methylumbelliferyl β-D-glucuronide (MUG) substrate (10 mM Tris-HCl, pH 8, 1 mM MUG (Gold Bio Tech) and 2 mM MgCl_2_), incubated for 30 min at 37°C, then added 100 μL of 0.2 M Na_2_CO_3_. The GUS activity was detected using the plate reader with the excitation filter at 355 nm and the emission filter at 460 nm.

### RNA extraction and quantitative real time-PCR

For quantitative real-time PCR, 1 μg of total RNA extracted with Trizol reagent (Invitrogen) was used for the first-strand cDNA synthesis by qScript Flex cDNA Synthesis kit (Quanta) as instructed by the manufacturer. The cDNA reaction mixture was diluted three times, and 2 μL was used as a template in a 15 μL qRT-PCR reaction (Quanta). PCR was performed after a preincubation at 95°C for 3 min followed by 40 cycles of denaturation at 95°C for 15 sec, annealing at 55°C for 15 sec, and extension at 72°C for 10 sec. All the reactions were performed in the BIO-RAD real-time PCR detection system. Each experiment was replicated three times. The primers used in RT-qPCR are listed in S2 Dataset.

### RNA-seq and data analysis

Three biological replicates of WT, *ati-2*, *topp1* mutant seeds were germinated in ½ MS liquid medium and grown up to 2-week-old with continuous shaking at 25°C. The seedlings were then treated with either mock or 3 hr of 50 μM ABA at room temperature. The total RNA was then isolated with Trizol reagent (Invitrogen) according to the manufacturer’s instruction and sequencing were carried out by Shanghai stress center.

Reads of RNA-seq were mapped to Arabidopsis reference genome (TAIR10) using TopHat using default parameters. Read count for each gene were obtained using feature Counts in subread. Feature differentially expressed (DE) genes were identified using DESeq (adjusted p-value < 0.05 and at least 4-fold change with ABA treatment). *TOPP1* or *AtI-2*-dependent genes were defined under the criteria: 1) genes were differentially expressed after ABA treatment in both WT and *topp1* (or *ati-2*) mutant; 2) gene expression level in *topp1* (or *ati-2*) mutant were at least 1.2 fold higher or lower than that in WT after ABA treatment. GO enrichment analysis were performed using WEGO [53].

### GUS staining

Germinating seeds or 2 or 3-week-old seedlings were incubated in GUS staining buffer (1 mM K_3_Fe(CN)_6_, 0.1% Triton X-100, 10 mM EDTA, 100 mM phosphate buffer (NaPO_4_, pH 7.0, and 2 mM X-Gluc) at 37°C for 16 h and the non-specific staining were removed with 50% ethanol. Tissue specific gene expression were observed by Leica EZ4 imaging system.

### Water loss measurement

Fully expanded adult leaves were excised from each genotype and left at room temperature, the fresh weight was recorded at the indicated time point. Water loss rate was expressed as the percentage of initial fresh weight. The experiment was repeated at least three times.

## Supporting Information

S1 Fig The interaction between TOPP2 and SnRK2s in Y2H. TOPP2 was fused to GAL4-activating domain (AD) and SnRK2s were fused to the GAL4-DNA binding domain (BD). Y2H assay was performed as described in Fig 1.
(JPG)

S2 Fig The interaction between TOPP1/AtI-2 and SnRK2.6 in pull down assay. Equal amount of purified GST-TOPP1, GST-AtI-2 or negative control GST only was incubated with MBP-SnRK2.6 in amylose resin. The elutes were resolved by SDS-PAGE and stained by coomassie blue.
(TIF)

S3 Fig Phosphatase activity of TOPP1 *in vitro*. (A) Recombinant protein TOPP1 showed phosphatase activity which could be suppressed by AtI-2 *in vitro*. (B) TOPP1 directly dephosphorylates SnRK2.6. (C) His-PYL11 inhibits TOPP1 activity. The phosphatase activity of TOPP1 was determined by a colorimetric assay using the substrate p-nitrophenyl phosphate (pNPP). Error bars indicate SD (*n* = 3).
(TIF)

S4 Fig TOPP1 and AtI-2 suppress SnRK2.2 and SnRK2.3 signaling. (A) SnRK2.2 mediated induction of *RD29B-LUC* was significantly reduced in the presence of TOPP1 and/or AtI-2. (B) The SnRK2.3 mediated induction of *RD29B-LUC* was inhibited by TOPP1 and/or AtI-2. The experiments were repeated at least three times and the data present are the mean values ±SD (n = 3).
(TIF)

S5 Fig Isolation of homozygous T-DNA insertion lines of *TOPP1* and *AtI-2*. T-DNA insertion homozygous mutants were identified by *TOPP1*, *AtI-2* gene-specific primers with LBb1.3 (LB) as indicated in each panel of (A) and (B). The gene expressions of *TOPP1* and *AtI-2* were determined by RT-qPCR in their mutants and transgenic plants (C). The relative transcription levels were normalized to *Act 2*.
(JPG)

S6 Fig ABA inhibition of seedling growth of wild type, *topp1* and *ati-2*. Photographs were taken at 7 days after seedling transfer to MS plates without or with 10 μM ABA. Three-day-old seedlings with equal root lengths were transferred. The experiments were repeated at least three times with similar results.
(TIF)

S7 Fig Salt sensitivity of *topp1* and *ati-2* mutants and overexpression lines. (TIF)

S8 Fig Water loss from detached leaves of wild-type, *topp1*, *ati-2* and *TOPP1*/*AtI-2* overexpression lines. The water loss percentage of wild type, *topp1*, *ati-2* (A) and *TOPP1*/*AtI-2* overexpression lines (B) was expressed as the percentage of initial fresh weight. Data presents average values ±SD from 20 leaves for each of three independent experiments.
(TIF)

S1 Dataset Differentially expressed ABA responsive genes that were significantly increased or decreased in *topp1* and *ati-2* mutants compared with WT. (XLSX)

S2 Dataset List of primers used in this study. (XLSX)
