# Supplementary figures and images for: Type One Protein Phosphatase 1 and Its Regulatory Protein Inhibitor 2 Negatively Regulate ABA Signaling
Source: PLoS Genet. 2016 Mar 4;12(3):e1005835. doi: 10.1371/journal.pgen.1005835 (PMC4778861; doi:10.1371/journal.pgen.1005835)

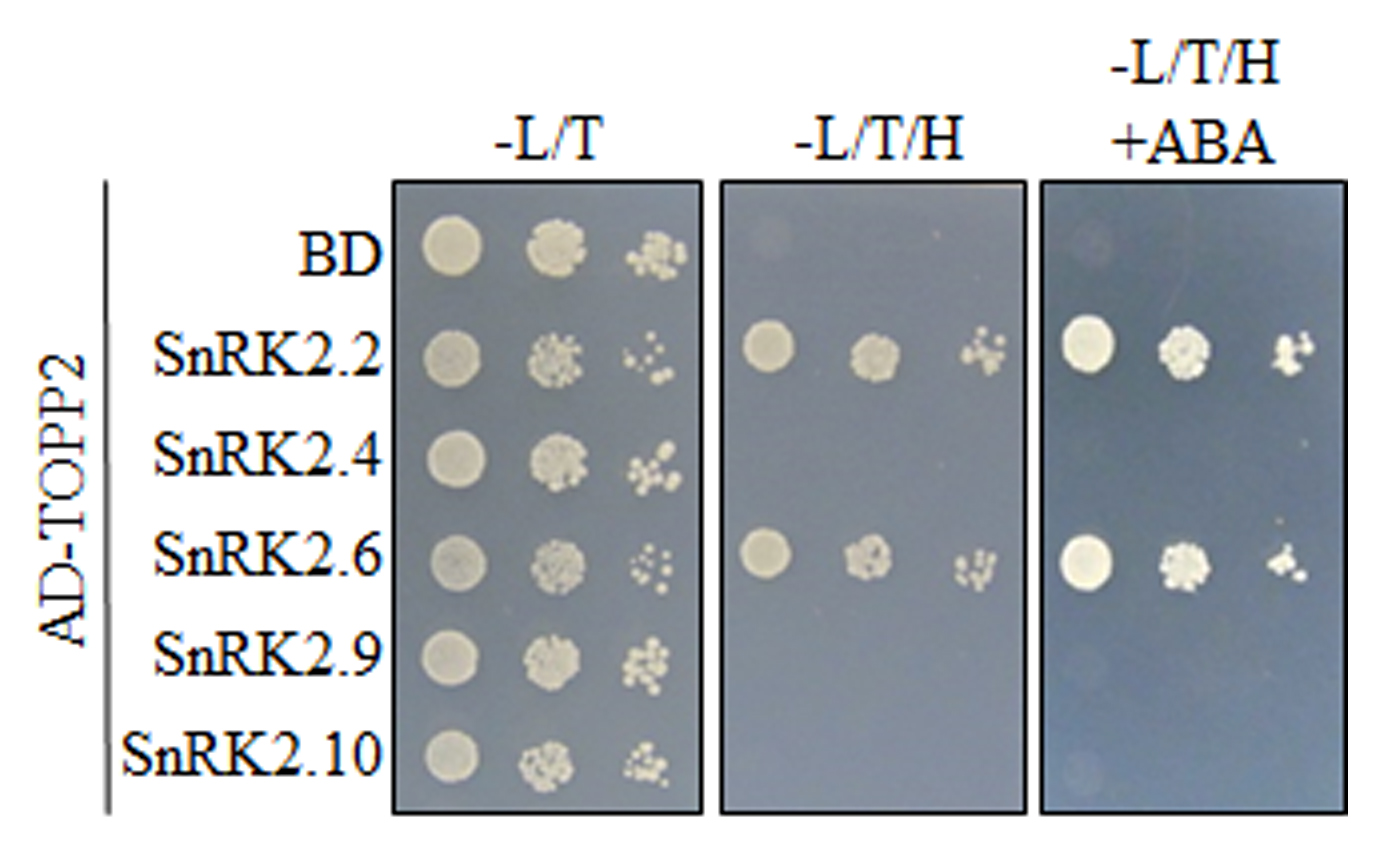

Supplement: S1 Fig — TOPP2 was fused to GAL4-activating domain (AD) and SnRK2s were fused to the GAL4-DNA binding domain (BD). Y2H assay was performed as described in Fig 1. (JPG) [file pgen.1005835.s001.jpg]

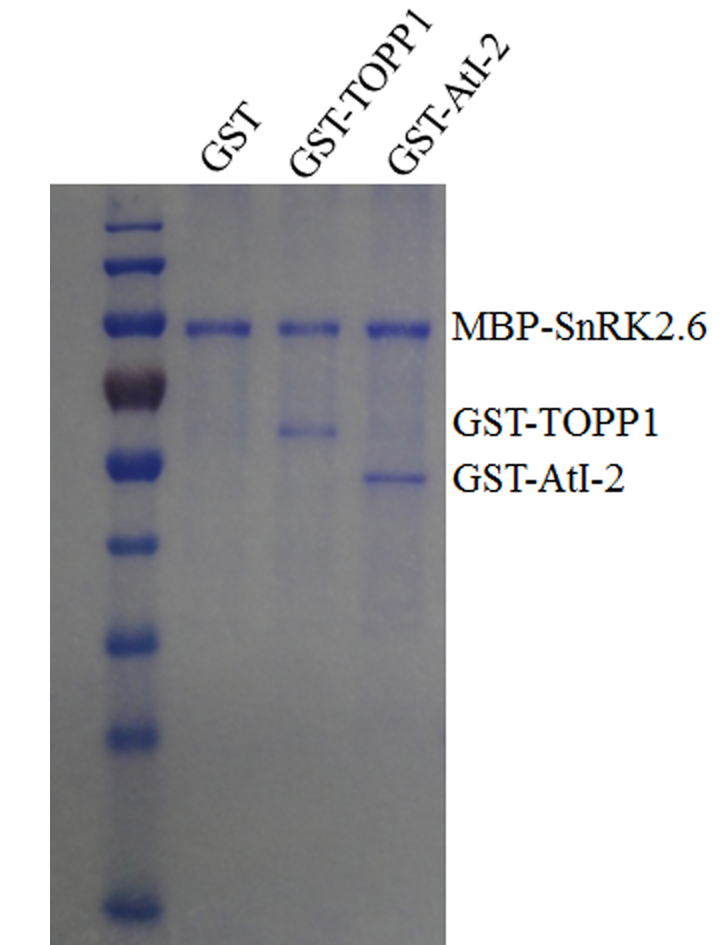

Supplement: S2 Fig — Equal amount of purified GST-TOPP1, GST-AtI-2 or negative control GST only was incubated with MBP-SnRK2.6 in amylose resin. The elutes were resolved by SDS-PAGE and stained by coomassie blue. (TIF) [file pgen.1005835.s002.tif]

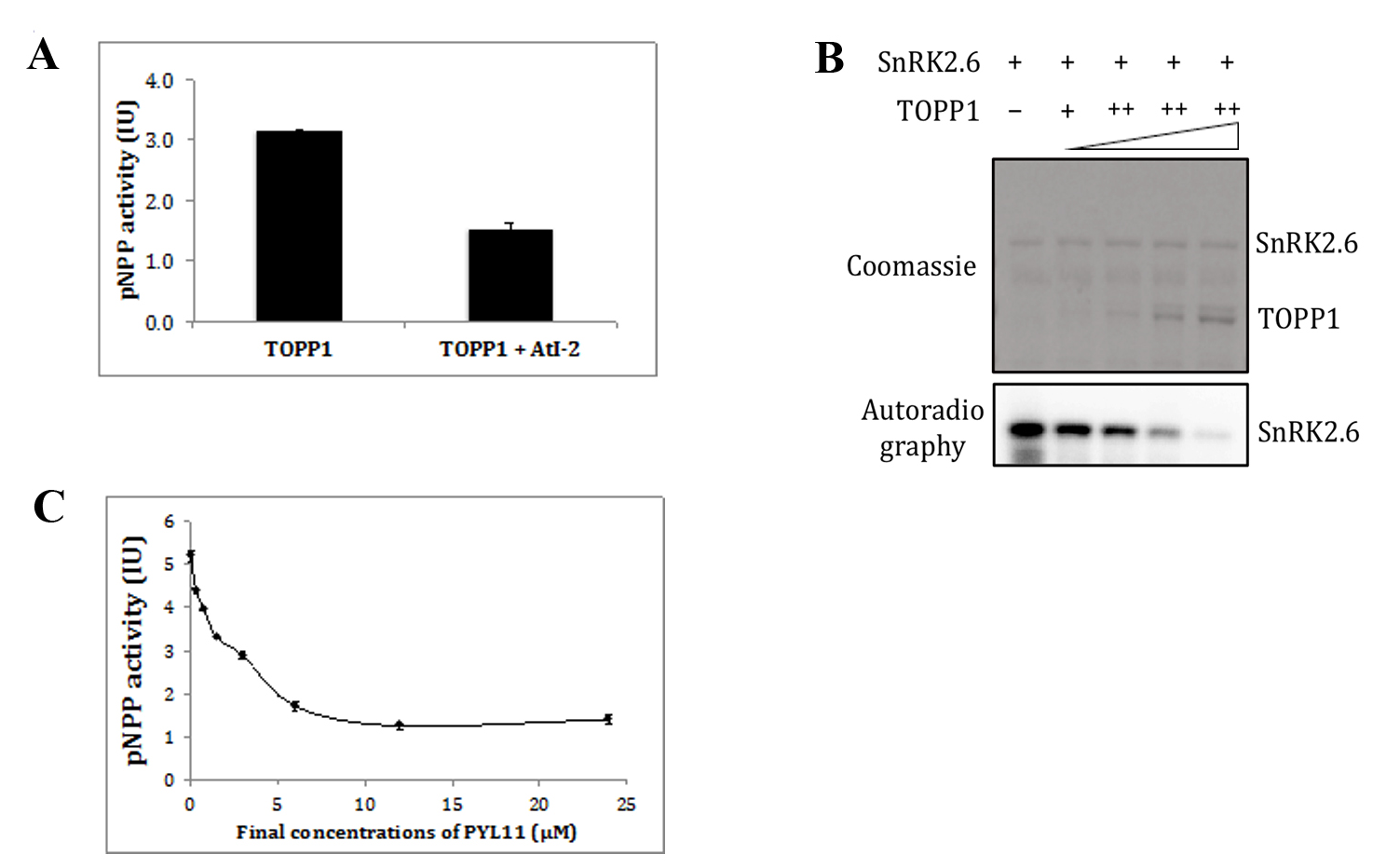

Supplement: S3 Fig — (A) Recombinant protein TOPP1 showed phosphatase activity which could be suppressed by AtI-2 in vitro. (B) TOPP1 directly dephosphorylates SnRK2.6. (C) His-PYL11 inhibits TOPP1 activity. The phosphatase activity of TOPP1 was determined by a colorimetric assay using the substrate p-nitrophenyl phosphate (pNPP). Error bars indicate SD (n = 3). (TIF) [file pgen.1005835.s003.tif]

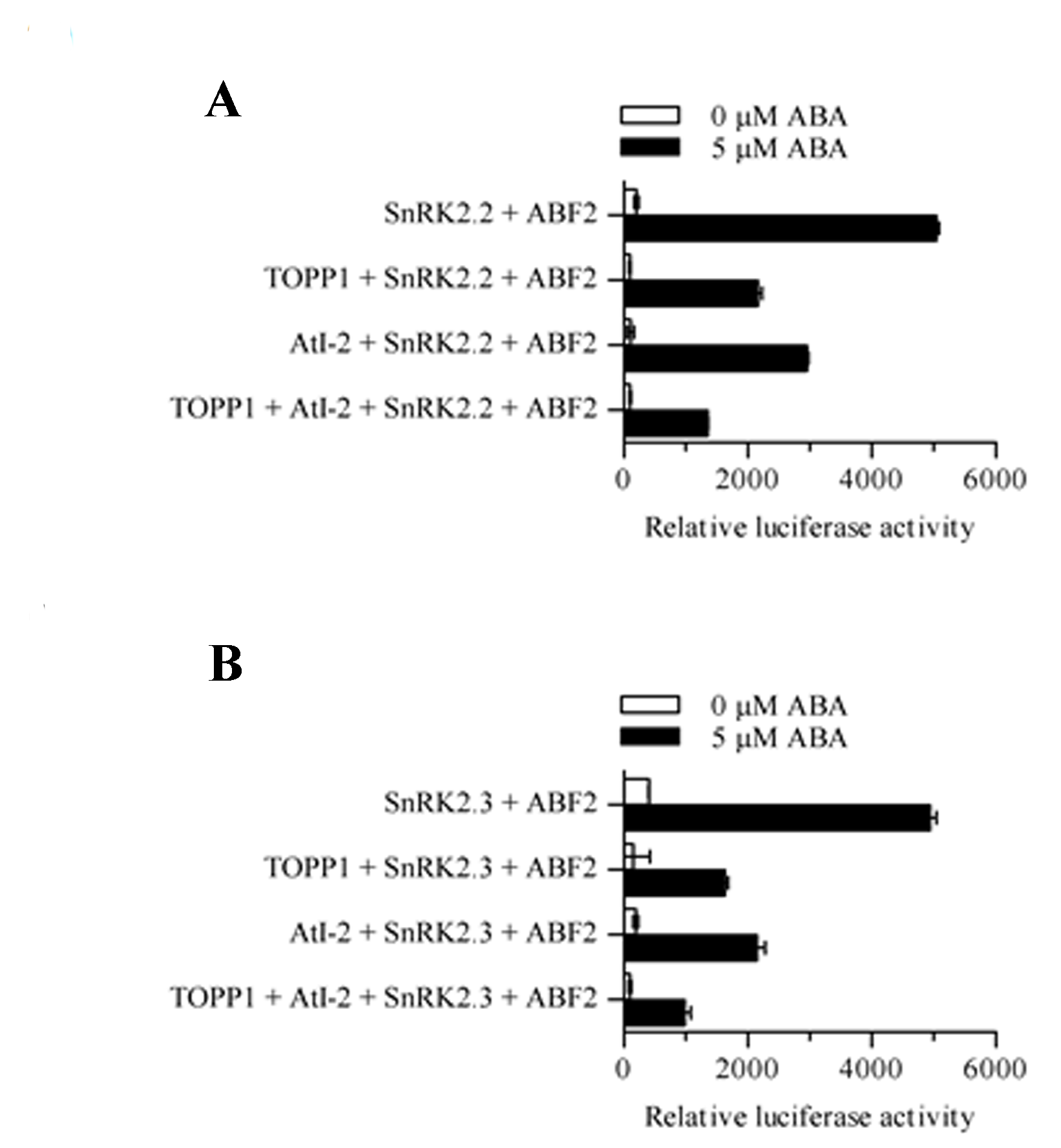

Supplement: S4 Fig — (A) SnRK2.2 mediated induction of RD29B-LUC was significantly reduced in the presence of TOPP1 and/or AtI-2. (B) The SnRK2.3 mediated induction of RD29B-LUC was inhibited by TOPP1 and/or AtI-2. The experiments were repeated at least three times and the data present are the mean values ±SD (n = 3). (TIF) [file pgen.1005835.s004.tif]

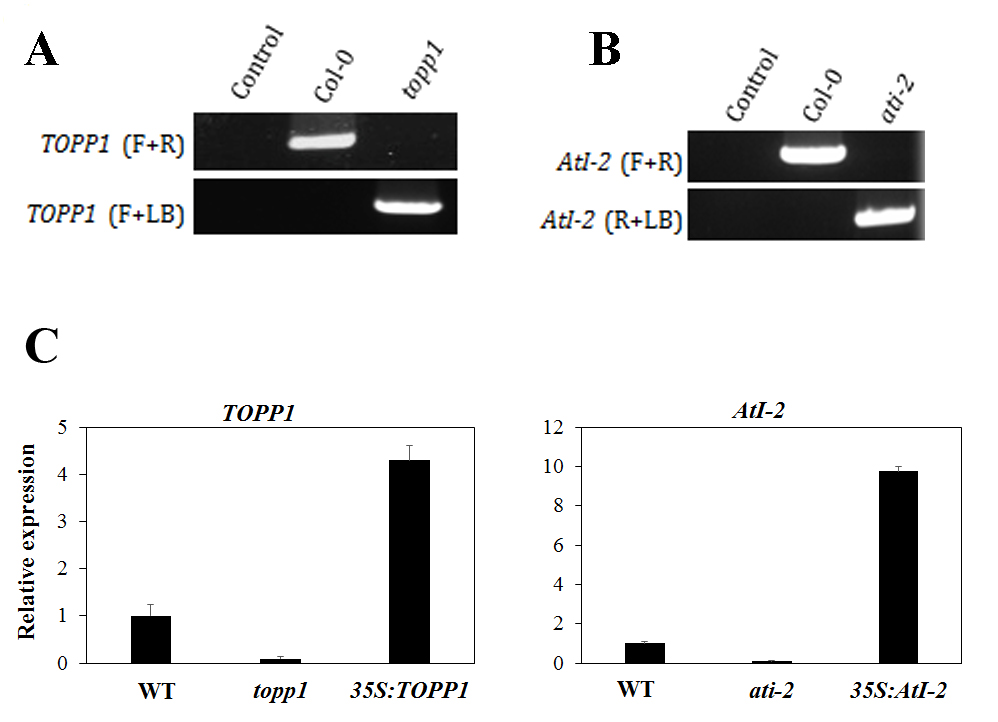

Supplement: S5 Fig — T-DNA insertion homozygous mutants were identified by TOPP1, AtI-2 gene-specific primers with LBb1.3 (LB) as indicated in each panel of (A) and (B). The gene expressions of TOPP1 and AtI-2 were determined by RT-qPCR in their mutants and transgenic plants (C). The relative transcription levels were normalized to Act 2. (JPG) [file pgen.1005835.s005.jpg]

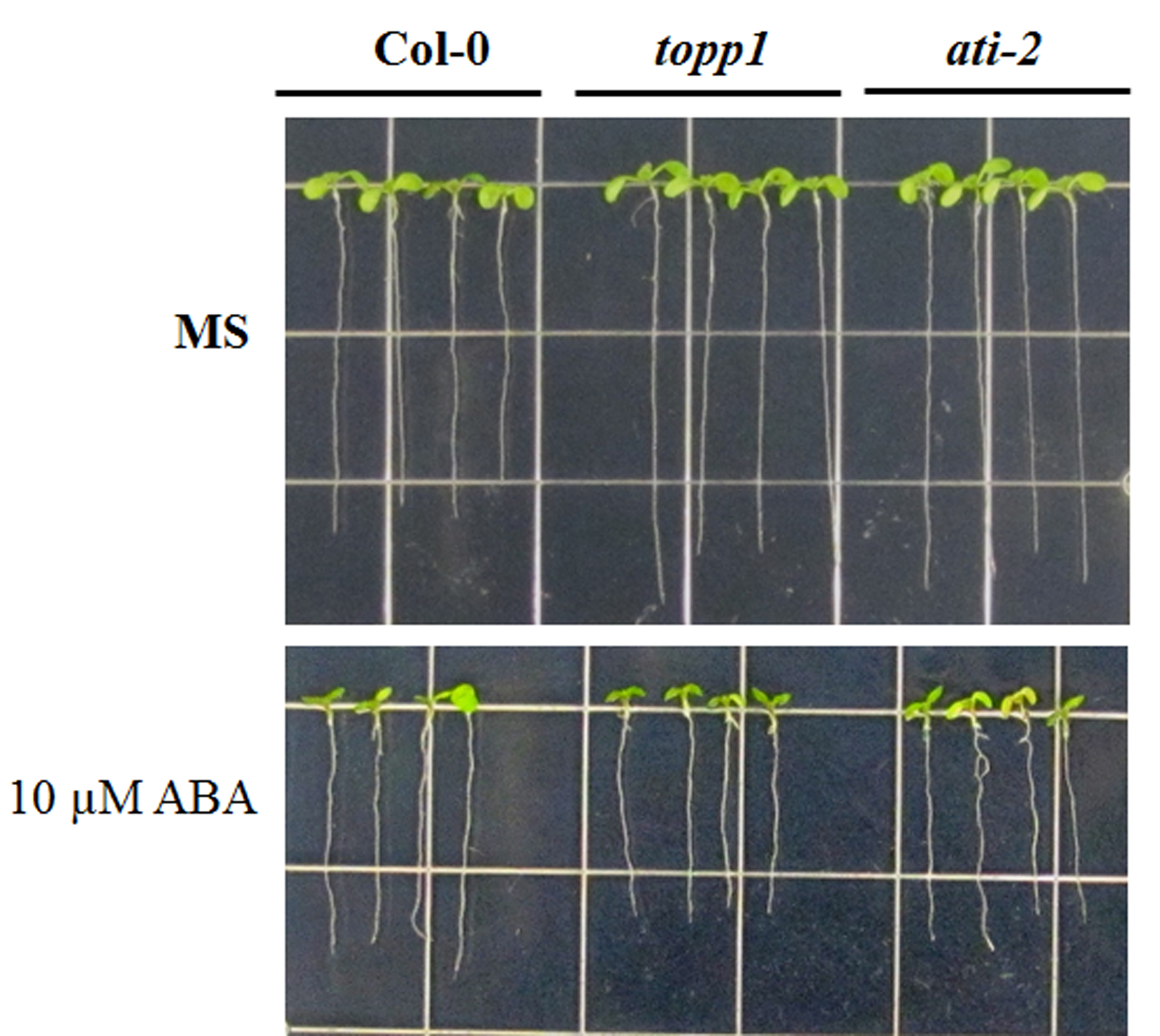

Supplement: S6 Fig — Photographs were taken at 7 days after seedling transfer to MS plates without or with 10 μM ABA. Three-day-old seedlings with equal root lengths were transferred. The experiments were repeated at least three times with similar results. (TIF) [file pgen.1005835.s006.tif]

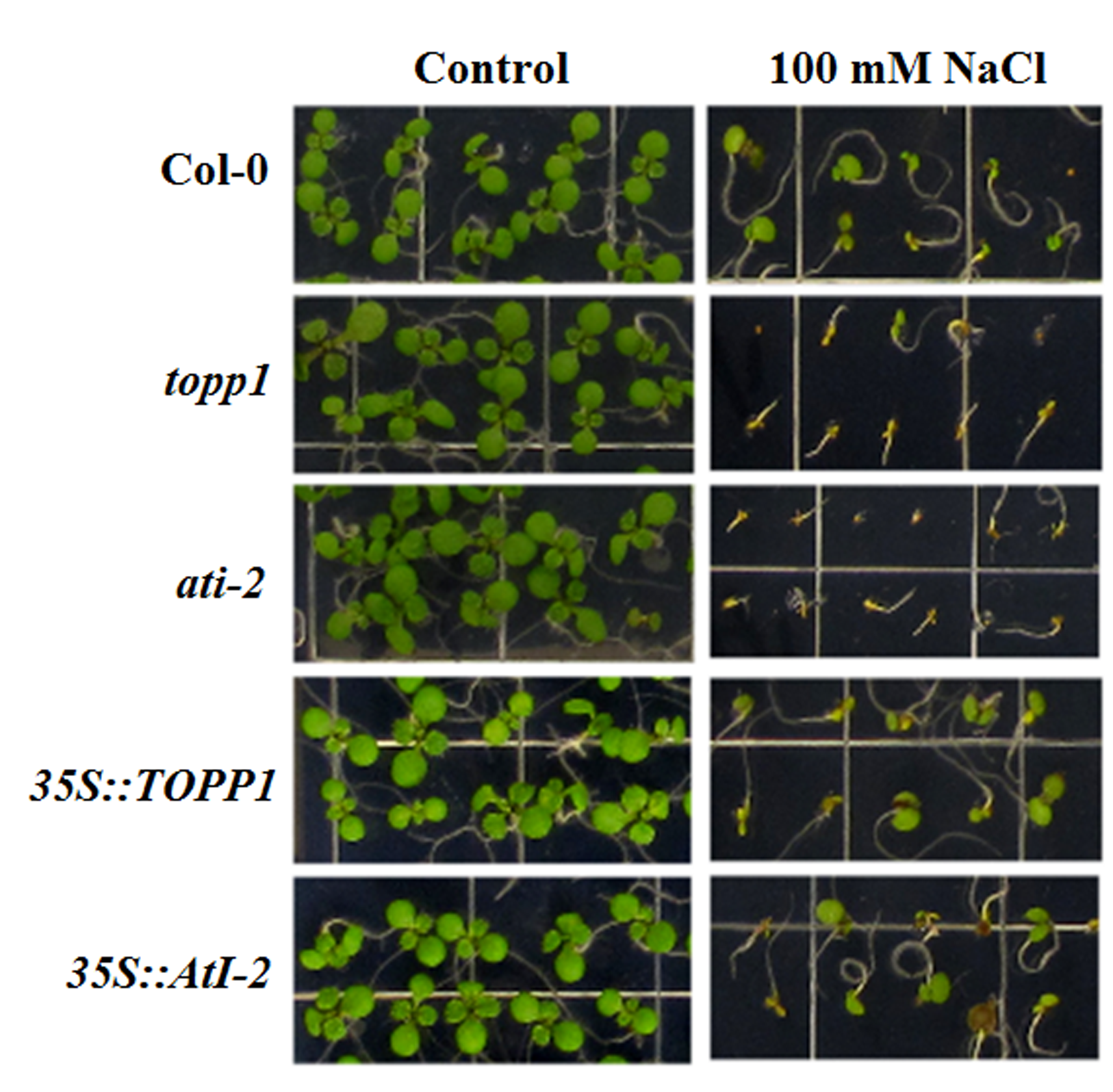

Supplement: S7 Fig — (TIF) [file pgen.1005835.s007.tif]

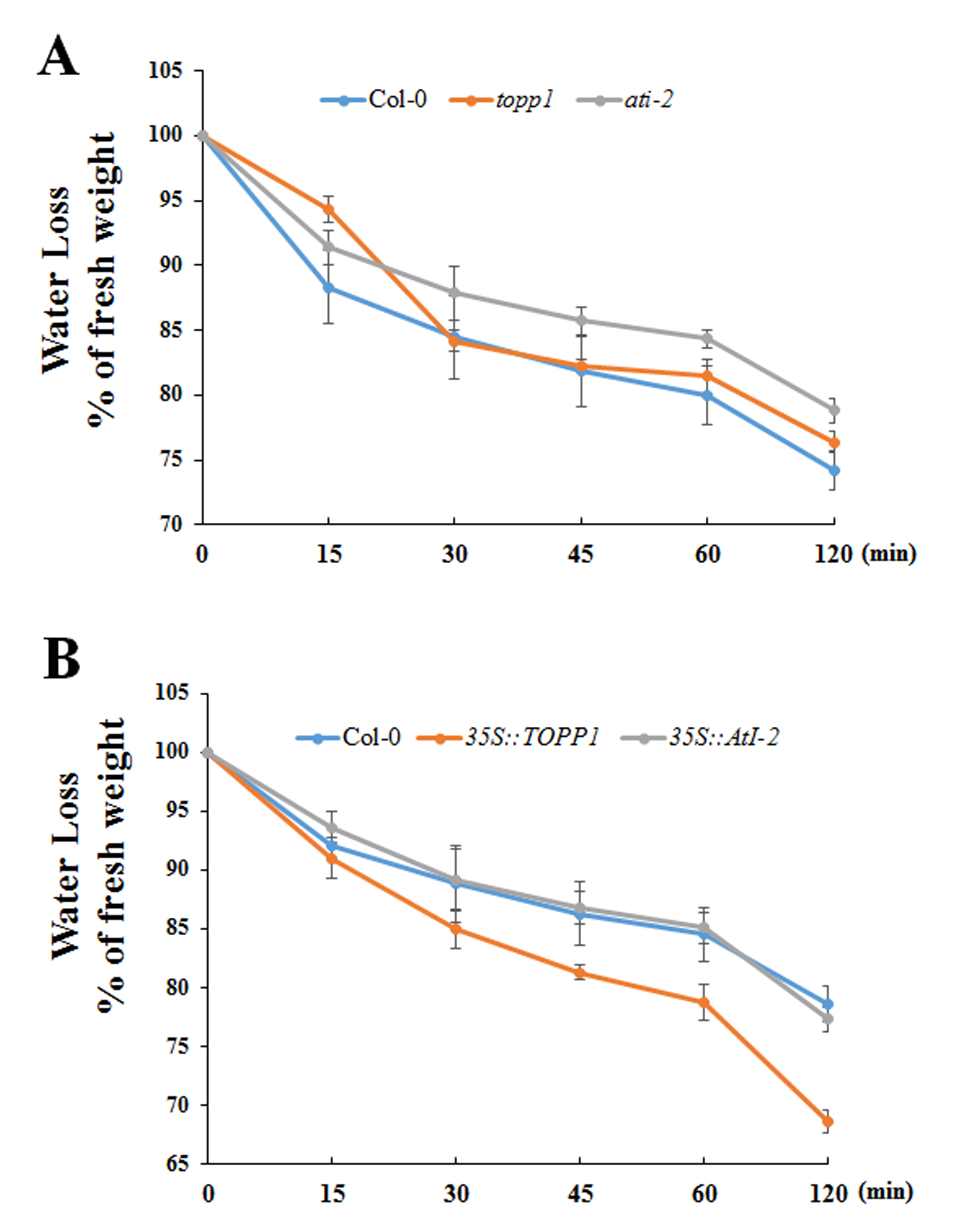

Supplement: S8 Fig — The water loss percentage of wild type, topp1, ati-2 (A) and TOPP1/AtI-2 overexpression lines (B) was expressed as the percentage of initial fresh weight. Data presents average values ±SD from 20 leaves for each of three independent experiments. (TIF) [file pgen.1005835.s008.tif]
